# Updated annotation and meta-analysis of *Brugia malayi* transcriptomics data reveals consistent transcriptional profiles across time and space with some study-specific differences in adult female worm transcriptional profiles

**DOI:** 10.1371/journal.pntd.0012511

**Published:** 2024-09-26

**Authors:** Christopher I. Holt, Julie C. Dunning Hotopp

**Affiliations:** 1 Institute for Genome Sciences, University of Maryland School of Medicine, Baltimore, Maryland, United States of America; 2 Department of Microbiology and Immunology, University of Maryland School of Medicine, Baltimore, Maryland, United States of America; 3 Greenebaum Cancer Center, University of Maryland School of Medicine, Baltimore, Maryland, United States of America; Beijing Friendship Hospital, Capital Medical University, CHINA

## Abstract

Genomics, transcriptomics, and proteomics have significantly advanced our understanding of obligately host-associated microbes, where interrogation of the biology is often limited by the complexity of the biological system and limited tools. This includes the causative agents of many neglected tropical diseases, including filarial nematodes. Therefore, numerous transcriptomics studies have been undertaken on filarial nematodes. Most of these transcriptomics studies focus on *Brugia malayi*, which causes lymphatic filariasis and is a laboratory model for human filarial disease. Here, we undertook a meta-analysis of the publicly available *B*. *malayi* transcriptomics data enabling the direct cross comparison of samples from almost a dozen studies. This reanalysis highlights the consistency of transcriptomics results across many different studies and experimental designs from across the globe for over a decade of research, across many different generations of a sequencing technology, library preparation protocols, and differential expression tools. Males and microfilariae across samples had similar expression profiles. However, female samples were clustered into two differential expression patterns that were significantly different from one another. Largely, we confirm previous results for all studies reanalyzed including tissue-specific gene expression and anti-*Wolbachia* doxycycline treatment of microfilaria. However, we did not detect previously reported differential expression upon *in vitro* or *in vivo* treatment with ivermectin, albendazole, and DEC, instead identifying a consistent lack of transcriptomic change upon exposure to these anthelminthic drugs. Updated annotation has been provided that denotes poorly supported genes including those overlapping rRNAs.

## Author summary

Numerous previous studies have measured all of the mRNA in *Brugia malayi* under a variety of conditions. Here, we collectively analyzed all data from those previous studies to gain new insights, which can be facilitated by the additional comparisons of this type of meta-analysis. Importantly, this meta-analysis demonstrates that worms that are shipped to various locations and handled by different laboratories are largely the same. The exception are adult females, where two profiles are apparent that are vastly different, which could lead to differing results in laboratory experiments. We propose that shipping dates and conditions be provided in manuscripts and that a transcriptome profile be collected in future laboratory studies to benchmark worms. In addition, the inclusion of more worm samples reveals that differences between worm shipments and batches is greater than the difference observed upon treatment with anthelminthic agents (ivermectin, albendazole, and DEC) under the conditions examined in the original studies. During this work, we identified some regions of the genome where genes were misidentified that affect RNA-based analysis and provide an updated annotation file to facilitate their removal in future studies.

## Background

Lymphatic filariasis is a mosquito-borne neglected tropical disease that affects people living in the equatorial regions of Latin America, Africa, and Southeast Asia [1]. The disease is currently estimated to afflict 67–120 million people across 72 countries, and almost 900 million people are at risk of infection [2–4]. Lymphatic filariasis in humans is caused by three filarial nematodes: *Wuchereria bancrofti* (~90% of cases), *Brugia malayi* (~10% of cases), and *Brugia timori* (<1% of cases) [2, 5]. *W*. *bancrofti* is found in equatorial regions of the Americas, Africa, and Southeast Asia whereas *B*. *malayi* and *B*. *timori* are restricted to Southeast Asia [1]. The L3 larval form of these nematodes are transmitted to vertebrate animals by the bite from *Anopheles*, *Aedes*, *Culex*, *Mansonia*, and *Ochlerotatus* mosquitoes [6]. During the life cycle of the nematode in the vertebrate host, the nematodes will migrate through the vascular system and into the lymphatics, where molting into the adult worm occurs, and microfilariae are subsequently released into the bloodstream where they are picked up by mosquitoes taking a blood meal [7]. Infection with Bancroftian or Brugian filariasis can lead to the development of human lymphedema, usually caused from damaged lymphatics due to the death of the worms and leading to the retention of fluid generally in the arm, leg, or groin [7,8]. As an invertebrate animal, *B*. *malayi* has its own complex morphology [9] that can be exploited for disease prevention and/or treatment, including vaccine development [10]. Currently, the transmission of lymphatic filariasis is combatted with preventative chemotherapies with drugs such as albendazole (Alb), diethylcarbamazine (DEC), and ivermectin (IVM), which are administered as part of a mass drug administration program aimed at eliminating disease [6]. *B*. *malayi*, along with other medically important filarial nematodes, also have an obligate *Wolbachia* endosymbiont [11], which has become a promising drug target since antibiotics will clear adult worms [12–17].

Since 2011, the transcriptome of *B*. *malayi* has been studied to examine the effect of drug treatments, changes over the life cycle, effect of *in vitro* culturing, and site-specific gene expression [18–29]. Published results rely on different analysis pipelines and differential expression detection methods, making them difficult to compare directly. Therefore, we reanalyzed all existing datasets with a unified pipeline consistent with RNA-Seq best practices [30] similar to a meta-analysis of *Wolbachia* transcriptome data [31]. This was supplemented with re-analysis using the original study design, which is frequently pairwise, with the more vigorously filtered data and with the most recent and stringent statistical tests (**S1 File**).

## Methods

### Data alignment and counts

FASTQ files were downloaded from the Sequence Read Archive (SRA) (**Table 1**) using SRAToolKit v2.10.9 and preprocessed using fastp v0.22.0 with default options [32]. These reads were mapped to a combined *B*. *malayi* reference genome downloaded from Wormbase version WS276 that included the nuclear genome, the AF538716.1 *B*. *malayi* mitochondria genome, and the ASM838v1 *Wolbachia* genome [33,34] using HISAT2 v2.1.0 (max-intronlen 5000) [35], sorted by position, merged as necessary (**S1 Table**), and indexed using samtools v1.9 [36]. Read counts were generated per gene feature, as defined in the GFF file, using HTSeq-Count v0.12.4 (python 3.8.2) in union mode [37]. The strandness option used for HISAT2 and HTSeq was determined from the RNA-Seq library preparation mentioned in the original paper and confirmed with Salmon [38] (**Table 1**). Summary statistics, including total number of reads, total number of primary aligned reads, and number of duplicate reads, were calculated for each bam file using samtools [36] with duplicates counted using picardtools MarkDuplicates [39] (**S1 Table**).

**Table 1:** Studies in this *B*. *malayi* Meta-Analysis.

Study	Reference	PMID	Year Published	BioProject	SRA Project	Library	Sequencing Platform	Reads	Strand^1^
A Deep Sequencing Approach to Comparatively Analyze the Transcriptome of Lifecycle Stages of the Filarial Worm, *Brugia malayi*	[18]	22180794	2011	PRJEB2709	ERP000948	Illumina mRNA Seq Kit	Illumina GA IIx	2x54bp PE^2^	None^3^
The Effects of Ivermectin on *Brugia malayi* Females *In Vitro*: A Transcriptomic Approach (Study I)	[19]	27529747	2016	PRJNA303987	SRP066610	NEBNext Ultra	Illumina MiSeq	2x150bp PE	None
The Effects of Ivermectin on *Brugia malayi* Females *In Vitro*: A Transcriptomic Approach (Study II)	[19]	27529747	2016	PRJNA303986	SRP066611	NEBNext Ultra	Illumina MiSeq	2x150bp PE	None
The Effect of *In Vitro* Cultivation on the Transcriptome of Adult *Brugia malayi*	[20]	26727204	2016	PRJNA294426	SRP063061	NEBNext Ultra	Illumina MiSeq	1x150bp SE^4^	None
Characterization of innate immunity genes in the parasitic nematode *Brugia malayi*	[21]	27110057	2016	PRJNA329497	SRP078934	NEBNext Ultra	Illumina MiSeq	2x150bp PE	None
Defining *Brugia malayi* and *Wolbachia* symbiosis by stage-specific dual RNA-Seq	[22]	28358880	2017	PRJNA344486	SRP090644	NEBNext Ultra	Illumina HiSeq2500	2x150bp PE	None
Effects of diethylcarbamazine and ivermectin treatment on *Brugia malayi* gene expression in infected gerbils (*Meriones unguiculatus*)	[23]	33777408	2019	PRJNA388112	SRP108080	TruSeq Stranded	Illumina NexSeq500	2x150bp PE	RF
Drug Repurposing of Bromodomain Inhibitors as Potential Novel Therapeutic Leads for Lymphatic Filariasis Guided by Multispecies Transcriptomics	[24,25]	31796568	2019	PRJNA294263	SRP068692	NEBNext Ultra Directional RNA	Illumina HiSeq2500	2x100bp PE	RF
Prediction pipeline for discovery of regulatory motifs associated with *Brugia malayi* molting	[26]	32574217	2020	PRJNA557263	SRP216772	NEBNext Ultra II	Illumina NextSeq500	2x150bp PE	None
Dual RNAseq analyses at soma and germline levels reveal evolutionary innovations in the elephantiasis-agent *Brugia malayi*, and adaptation of its *Wolbachia* endosymbionts	[27]	33406151	2021	PRJEB40568	ERP124221	ScriptSeq Complete Gold	Illumina	2x75bp PE	None^3^
*Wolbachia* depletion blocks transmission of lymphatic filariasis by preventing chitinase-dependent parasite exsheathment	[28]	35377795	2022	PRJNA772674	SRP343552	NEBNext Ultra Directional	Illumina HiSeq 4000	2x150bp PE	RF
Spatial transcriptomics reveals antiparasitic targets associated with essential behaviors in the human parasite *Brugia malayi*	[29]	35390105	2022	PRJNA548881	SRP201437	Clontech SMARTSeq v4 Ultra-Low Input	Illumina HiSeq2500	1x100bp SE	None^3^

^1^Strandedness option used in HISAT2 and HTSeq

^2^PE = paired end

^3^Strandedness was not reported, so ran as unstranded

^4^SE = single end.

### Filtering due to rRNA annotation issues

Eleven genes overlapping the rRNA were initially identified when troubleshooting the analysis pipeline. WBGene00220294 was not identified at that time and thus was not removed, but this should be examined in the future. These eleven genes are WBGene00228061, WBGene00268654, WBGene00268655, WBGene00268656, WBGene00268657, WBGene00221211, WBGene00220288, WBGene00220284, WBGene00268299, WBGene00228060, and WBGene00268300 (**S1 Fig**). This includes the five *B*. *malayi* genes that were removed in a previous analysis (WBGene00228061, WBGene00268654, WBGene00268655, WBGene00268656, and WBGene00268657) [24]. A gff file is provided where these eleven genes are removed (**S2 File**). We recommend that they be removed from analyses and ultimately the *B*. *malayi* annotation.

### Rarefaction curve and variance partition

To verify that there was a sufficient number of reads, a rarefaction curve of all replicates using all counts was generated using the package vegan v2.6–4 [40,41] on a per study basis (**S1 File**). Using variancePartition v1.26.0 and limma v3.52.4, a linear mixed model was applied to the raw counts data to assess the effect of various factors on gene variation [42–44]. Replicates that did not reach saturation in the rarefaction analysis were subsequently removed as were all single replicate samples that resulted from this removal.

### CPM filtering and differential expression analysis

Genes were filtered to only include those that met a counts-per-million (CPM) cut off value of 5 in a minimum number of samples equal to the smallest number of replicates in a group, and samples consisting of only one replicate were removed. CPM is the number of reads per gene divided by the total number of sequenced reads per sample multiplied by one million [24]. This allows for a threshold that is normalized for differences in the count magnitudes between samples. EdgeR v3.30.3 was used to identify differentially expressed genes with the quasi-likelihood negative binomial generalized log-linear (glmQLFit) model [45] (*p*-value < 0.05; FDR cutoff < 0.05 after Benjamini Hochberg correction). No additional log_2_-fold change cutoffs were applied. Using z-score normalized log_2_(TPM) values of the differentially expressed genes, a principal components analysis (PCA) plot and dendrogram were generated using FactoMineR v2.6 [46] and pvclust v2.2–0 [47], respectively. Heatmaps were generated with heatmap.3, including a z-score normalized heatmap of log_2_ transformed TPM values and an unnormalized heatmap using only the log_2_ transformed TPM values. The differential expression clusters were generated using WGCNA v1.71 [48].

### Statistical analysis and plotting

All differential expression analysis was performed in R v4.2.1 [49]. The rarefaction curve, PCA, and output of variancePartition were all plotted using the ggplot2 v3.4.0 package [50]. The package tidyverse v1.3.2 [51] was used for data processing, the package ggdendro v0.1.23 [52] was used to acquire the order of the pvclust dendrogram, and the package devtools [53] was used to download the heatmap.3 function. All scripts and commands have been uploaded to https://github.com/christopher-holt/bmalayi_meta_analysis and archived at https://zenodo.org/doi/10.5281/zenodo.13694246. They are available under the MIT license. Default settings were used unless otherwise specified or shown.

## Results

### Similarity of Expression through Time and Space: Life Cycle-Specific Gene Expression

Of a potential 244 *B*. *malayi* transcriptomics samples, 7 were removed due to low read counts or due to being a single replicate sample after removing low read count samples (**S1 Table**). The remaining 237 samples spanned over 11 years of research including different generations of sequencing technologies, mRNA enrichment and/or rRNA depletion methods, library construction methods, and study designs (**Tables 1 and S1**). The reads generated for two of the studies were single ended [20, 29] with the rest being paired end reads and only three being from strand-specific libraries [23,24,28] (**Table 1**). Some studies used poly(A) enrichment [18–21,24] while the others used rRNA depletion to enrich for mRNA [22,26,28] or did not specify [23,29]. Several generations of Illumina sequencing technology were used for these studies as well as different length reads (ranging from 2x54 bp paired-end reads [18] to 2x150 bp paired end reads [28]) (**Table 1**).

Out of the 10,988 genes that were considered, 1,261 genes were removed using the cpm filter, meaning that they had too few reads in far too many samples (**S2 Table**). These genes had fewer exons (p-value = 0.0005, Fisher Exact Test 2x85) with 133 (11%) single exon genes and 484 (38%) double exon genes. Defining hypothetical genes as genes without an InterPro description, there is an overrepresentation of hypothetical genes in the genes that were removed by the cpm filter (p-value < 2.2e-16, Fisher Exact Test). These genes are not evenly distributed across the genome (p-value = 0.0005, Fisher Exact Test 2x196) being over-represented on unplaced contigs relative to the chromosomes (p-value <0.00001, Fisher Exact test 2x2) with 25% of the genes removed with the cpm filter being on unplaced contigs despite only 6.7% of the genome being on unplaced contigs. Unplaced contigs include all the contigs that could not be placed in an autosome or chromosome X, and they include chromosome Y contigs, telomeres, and other repeats. Although we cannot rule out that these unexpressed genes are just not sufficiently expressed under these conditions, their position on unplaced contigs and the low number of exons per gene collectively suggests that these may be low quality gene annotations that should be examined further, and possibly removed. To facilitate future transcriptomics studies, these genes have been marked with “Note = poorly supported by transcriptomics data Holt et al” in the updated gff annotation file provided (**S2 File**).

**Fig 1:**
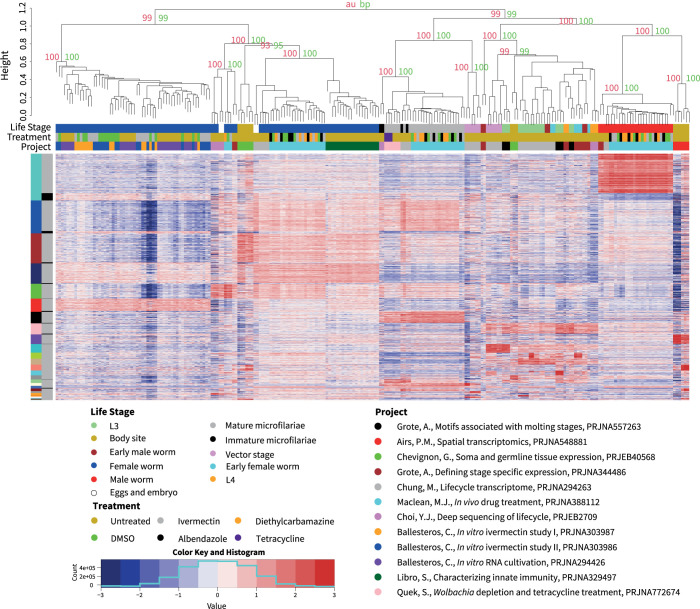
Heatmap of the 9,727 Differentially Expressed Genes in the Meta-Analysis. The dendrogram at the top of the heatmap was generated using pvclust. The red values are approximately unbiased (au) and the green are the bootstrap support (bp) values, both of which are generated by pvclust. The values shown are for illustrative purposes with the full dendrogram available in **S3 Fig**. The histogram at the bottom shows the distribution of all the z-score values in the heatmap. The heatmap uses a z-score normalization of log_2_(TPM) values for 9,727 differentially expressed genes between 237 samples reanalyzed from 12 projects. The legend at the top is broken into three sections: project color, if the sample was drug treated, and sample life stage. The left hand legend is broken into two sections: the outer section denotes the WGCNA cluster and the inner section denotes if cluster matches the main or inverse WGCNA cluster expression pattern. Samples are labeled with first author, title, and bioproject from [18–29].

The remaining 9,727 genes (88.5%) were identified as differentially expressed between the 237 samples, to a statistically significant level. These differentially expressed genes were clustered into 27 modules using WGCNA (**Figs 1 and S2**) while the samples were clustered using pvclust (**Figs 1 and S3**). Most of these WGCNA modules represent life cycle-specific gene expression, which has previously been described [18,21–24,26–28]. For example, the largest WGCNA cluster in the analysis of all data (cluster 1, turquoise) has 1,551 genes up-regulated and 287 genes downregulated in mature adult males (**S3 File**), including the major sperm proteins, papD-like functions, various protein kinase and phosphorylation, serine/threonine proteases, and phosphatases, as previously described [18,22,24] (**S4 File**).

### Study-specific differences in adult females

While life stage-associated expression patterns are overall shared across many datasets, there are study specific differences in the adult female samples. The adult female samples can be clustered into two well supported groups. The first group (female group 1) contains samples from three studies on ivermectin dose and RNA isolation [19, 20]. The second group (female group 2) contains samples from six studies that focused on the life cycle, immune challenge, germline, and exposure to ivermectin, albendazole, and diethylcarbamazine [18, 21–24, 27] (**Figs 1 and S3**). Studies in both groups used the same library construction protocols, sequencing technologies, and read lengths. The studies in both of the groups also included worms acquired from the NIAID FR3 [54] and included multiple studies where RNA was isolated from worms without shipping as well as isolation following shipping and acclimatization. The only life stage in common between these studies is adult females, precluding the examination of other life stages to help understand these differences. This is not merely an artifact of the z-score calculation on an uneven sampling distribution, since down sampling to 18 adult males, 18 microfilariae, 9 females from group 1, and 9 females from group 2 yielded the same result (**S4 Fig)**. The genes that were determined to be differentially expressed were enriched for genes without a GO term for cellular component, biological process, or molecular function and genes that lack an InterPro entry (**S5 Fig**). A comparison between selected samples of adult female worms reveals 156 significantly enriched terms and 7,347 differentially expressed genes. The differences also cannot be attributed to differences in the mitochondrial gene expression. We were unable to attribute the differences in gene expression to any source, other than that the worms in female group 1 were all from studies from a single lab, albeit at different times. There is concordance between differences in the adult female transcriptional profiles and differential expression in microfilariae. Given that these are relative expression profiles, it is difficult to discern the exact nature of these similarities and differences, but one possibility is that some adult females have an arrest in reproduction and are no longer producing microfilariae.

### Gene regulation in body sites

Genes differentially regulated in different worm body sites were detected in the meta-analysis. There are 384 differentially expressed genes that are upregulated in the adult female head and adult female body samples [29] relative to all other samples in the meta-analysis (cluster 9, purple), suggesting some study specific differences. The term head refers to the 0.6 mm of tissue above the vulva and contains most of the pharynx along with the nerve ring, the term body refers to the rest of the worm [29]. There are no statistically significant enriched functional terms for this cluster.

There are also 228 differentially expressed genes upregulated in only the body samples [29] (cluster 13, salmon) (**Figs 1 and S3**). However, using the meta-analysis alone yields an under-assessment of the differences between these body types. In a pairwise analysis using the same methods, we identified 6,256 differentially expressed genes between these head and body sites, that include 42 significantly enriched terms (**S1 File**). Enriched terms for genes up-regulated in the body sites include protein kinase and phosphorylation, phosphatidylinositol binding, and homeobox domain while terms up-regulated in the head include transthyretin-like and Pepsin inhibitor-3-like repeated domain terms.

**Fig 2:**
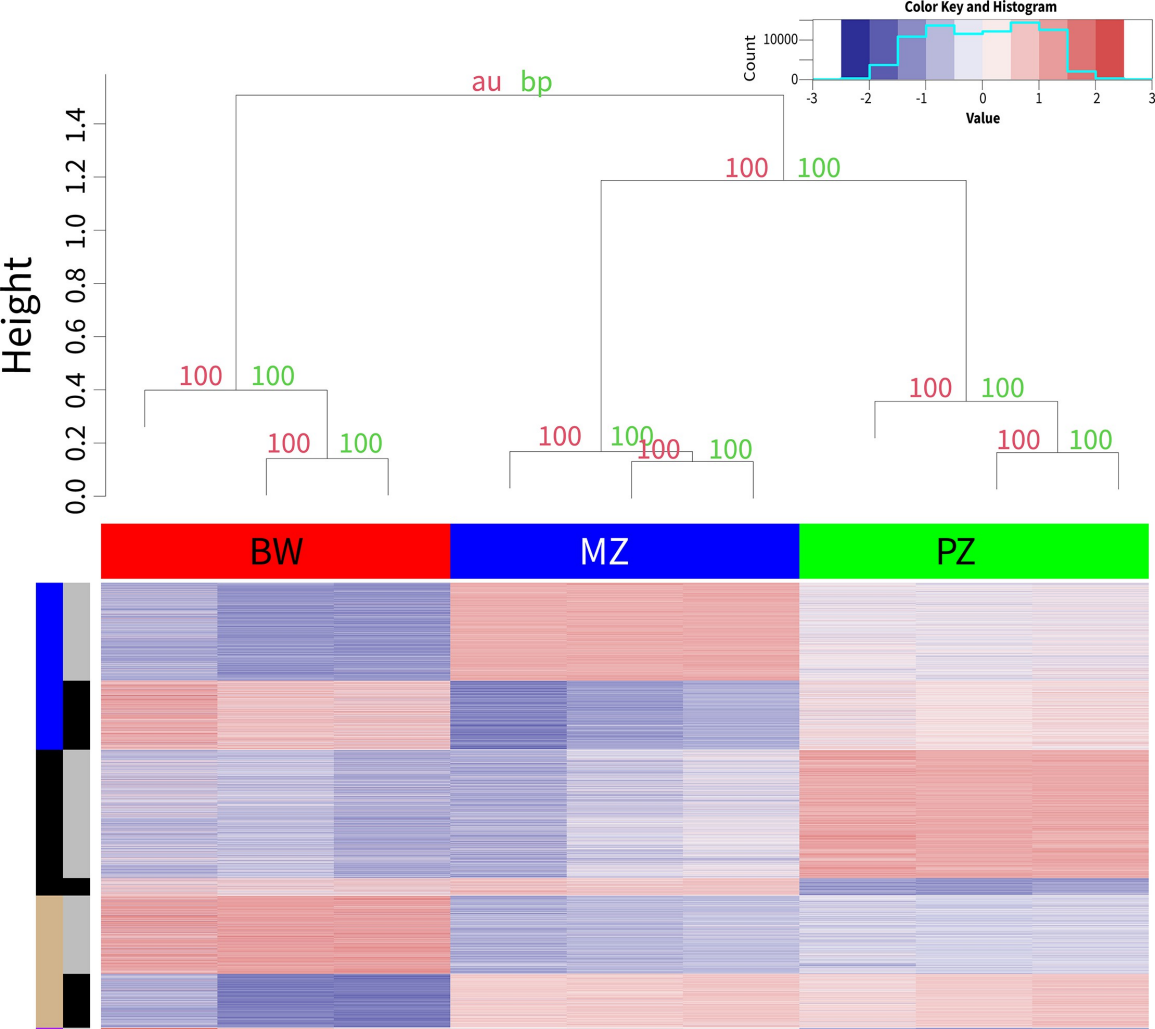
Differential Expression of Soma and Germline Samples. The dendrogram at the top of the heatmap was generated using pvclust. The red values are approximately unbiased (au) and the green are the bootstrap support (bp) values, both of which are generated by pvclust. The histogram at the bottom shows the distribution of all the z-score values in the heatmap. There are 7,191 differentially expressed genes between the proliferative zone (germline), meiotic zone (germline), and body wall (soma) samples. The heatmap shows a z-score normalization of log_2_(TPM) values for the 7,191 differentially expressed genes divided between three WGCNA clusters. The top legend denotes the site of tissue origin. The left hand legend is broken into two sections: the outer section denotes the WGCNA cluster and the inner section denotes if cluster matches the main (grey) or inverse (black) WGCNA cluster expression pattern.

There are similarities between the germline tissues (Proliferative Zone and Meiotic Zone) and eggs/embryos as well as mature adult females, while the soma samples are similar to worm stages early in development in the vertebrate animal. However, similar to above, this is likely an under-assessment of the differences since in a pairwise analysis of germline and soma samples using the same methods, we identified 7,191 differentially expressed genes with 52 statistically significant functionally enriched terms (**Fig 2 and S3 Table**) with 67% of the variance ascribed to sample variation (**Fig 2 and S1 File**).

### Condition-specific differential expression can be missed in the meta-analysis: Antibiotic treatment

**Fig 3:**
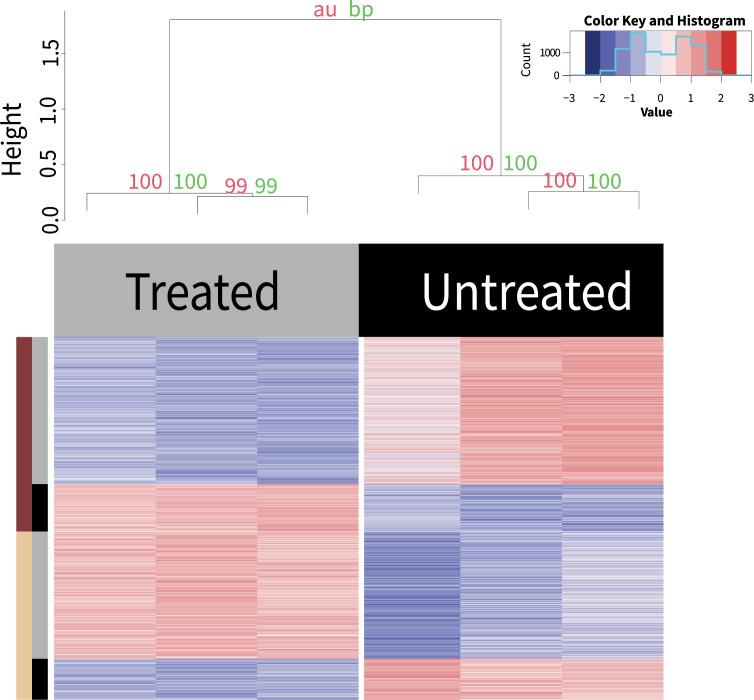
Differential Expression of Tetracycline Treatment. The dendrogram at the top of the heatmap was generated using pvclust. The red values are approximately unbiased (au) and the green are the bootstrap support (bp) values, both of which are generated by pvclust. The histogram at the bottom shows the distribution of all the z-score values in the heatmap. Between the two treatment groups, there are 1,423 DE genes sorted into two WGCNA clusters. A z-score normalization of log_2_(TPM) values was used for the heatmap. The top legend denotes the treatment groups. The left hand legend is broken into two sections: the outer section denotes the WGCNA cluster and the inner section denotes if cluster matches the main (grey) or inverse (black) WGCNA cluster expression pattern.

While life stage specific expression could be identified in the meta-analysis of all samples, other previously published expression profiles were not obvious. For example, the 1,079 previously identified differentially expressed genes associated with *Wolbachia* depletion using tetracycline in microfilariae [28] were not apparent. However, we were able to identify 1,423 differentially expressed genes in a pairwise comparison of the samples using the same filtering and glmQLFit model as in our unified meta-analysis (**Fig 3 and S4 Table**). Functional enrichment analysis of these differentially expressed genes revealed 12 significantly enriched terms, including IPR001579 for glycoside hydrolase chitinase active site and GO:0006032 for chitin catabolic process. This is consistent with the finding of the original study identifying chitinases as differentially expressed [28].

A similar result where an analysis of all samples in this unified analysis obfuscated results seen in a pairwise comparison was observed for the samples from across the L3-L4 molt (**S1 File**) [26]. This illustrates that differential expression for some comparisons is not readily apparent in the meta-analysis, likely due to clustering being driven by conditions with the largest number of samples/replicates, which in this case is life stage, and underscores the importance of targeted question/hypothesis-driven analyses.

### Condition-specific differential expression can be informed by the meta-analysis: The case of cultivation

When comparing the transcriptomes of freshly isolated *B*. *malayi* female worms to those held in culture media before RNA isolation [20], we were not able to resolve any differential expression in the large-scale analysis of all samples. However, in a pairwise re-analysis analogous to the original study design, we recapitulated the original results identifying 4,242 differentially expressed genes with 127 significantly enriched functional terms, including P-loop containing hydrolase, WD40-repeat-containing domain, EGF-like domain, ankyrin, zinc finger, protein kinase and protein phosphorylation (**S1 File**). We were not able to detect any differences between the cultured and non-cultured adult females worms in a differential expression analysis (**S6 Fig**).

### Filtering/preprocessing and model/statistics choice reveals a lack of differential expression upon anthelminthic exposure

In the unified meta-analysis, we were not able to identify any differential expression clusters for transcriptional changes upon exposure to anthelminthic compounds that did not target the *Wolbachia* endosymbiont, whether from *in vitro* exposure to ivermectin (**S1 File**) [19] or *in vivo* exposure to ivermectin, albendazole, and diethylcarbamazine (**S1 File**) [23]. Using the filtered data and the glmQLFit model did not reveal significant differential expression, suggesting that treated worms were not transcriptionally different from untreated worms.

**Fig 4:**
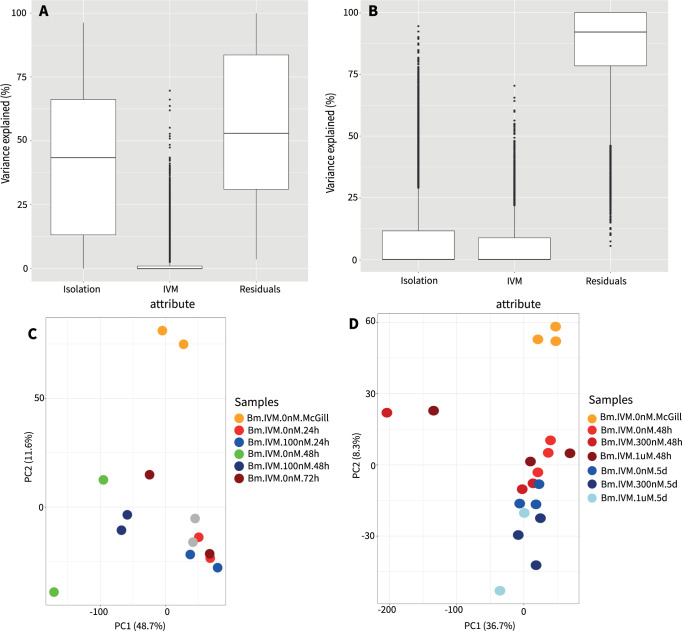
Lack of Differential Expression Upon Ivermectin Treatment. The linear mixed model and PCA plots show a lack of effect from Ivermectin treatment [19]. The linear mixed model was generated using all counts. The PCA plots were generated using z-score normalized log_2_(TPM) values for genes where the sum of the TPM values for each gene is greater than 0. **(A)** linear mixed model for study I (PRJNA303987) **(B)** linear mixed model (PRJNA303986). **(C)** Principal Components Analysis for Study I (PRJNA303987). The samples are colored based on IVM dosage and time period. **(D)** Principal Components Analysis for Study II (PRJNA303986). The samples are colored based on IVM dosage and time period.

The published analysis (Bioproject PRJNA303987, [19]) of worms exposed to 100 nM ivermectin with sampling every 24 h over 72 h used an exact test in EdgeR [45,55]. Treated samples were compared to untreated worms at the same time point, with 34 differentially expressed genes detected after 24 h of exposure, 421 differentially expressed genes after 48 h, and 15 differentially expressed genes after 72 h group [19]. When we apply the same exact test in EdgeR to pairwise comparisons using the filtered data we used here, we identify 3 differential expressed genes for 24 h and 72 h and 118 differential expressed genes for 48 h. This suggests that the filtering removes some of the genes originally identified as differentially expressed. Our unified analysis method on all datasets cannot use the exact test, as the exact test is only suitable for pairwise comparisons. When we apply the glmQLFit model using the filtered data, we identified no differential expressions at 24, 48, or 72 h. This illustrates that both the filtering and pre-processing of the data as well as the glmQLFit model used for statistical testing significantly influence the results. The glmQLFit model is expected to be more conservative, reducing Type I errors (thus minimizing false positive) by providing more robust error rate control [45,55]. In addition, the linear mixed model from variancePartition shows that residuals are the primary driver of separation, which is confirmed in a principal component analysis (**Fig 4A and 4B**), suggesting that the transcriptomes of treated and untreated samples are either not significantly different or that the study is under powered in the ability to detect weaker transcriptional differences given the biological variation in the system.

The second published analysis (Bioproject PRJNA303986, [19]) of worms exposed to 300 nM and 1 μM ivermectin to time matched controls at 48 h and 120 h, also used an exact test for the differential expression analysis EdgeR [45,55]. Across the four pairwise comparisons, they identified 68–271 differentially expressed genes using the exact test. However, using the pre-processing/filtering and glmQLFit model in the unified analysis, we did not identify any differentially expressed genes in any pairwise comparisons. This suggests that filtering and pre-processing alone led to the decrease in the detection of differentially expressed genes. The linear mixed model from variancePartition shows that residuals are the primary driver of separation, not the isolation time or the ivermectin concentration, which is confirmed in a principal component analysis (**Fig 4C and 4D**).

A third study (BioProject PRJNA388112, [23]) examined the effect of drug treatment (Albendazole, Diethylcarbamazine, Ivermectin or a control DMSO) and exposure time (one- or seven-days post treatment) on different life stages (adult male, adult female, and microfilariae). Using EdgeR glmQLFit model [45,55], including all 68 samples, we identified 9,350 genes that were differentially expressed which a dendrogram and PCA reveal is largely based on life stage and not based on drug exposure (**S1 File**). Using a linear mixed model from variancePartition grouping by life stage, treatment, and exposure, life stage (76.5%) and residuals (22.4%) account for almost all the differences in expression with treatment and exposure accounting for only a small amount of the variation (0% and 0.04% respectively) (**S1 File**). When the samples were separated by life stage and re-analyzed, no differentially expressed genes were identified (**S1 File**). This suggests that there is no differential expression upon treatment.

### Drug target gene expression

**Fig 5:**
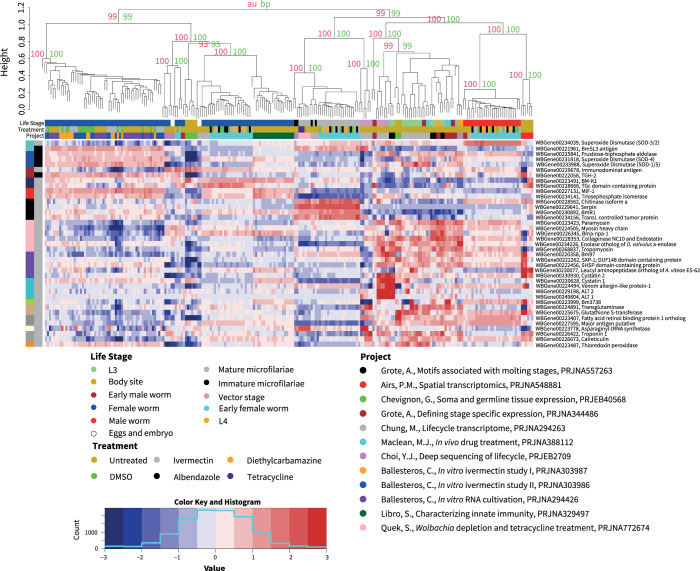
Expression of Candidate Drug Target Genes. Differential expression of potential drug target genes. The genes and clusters were pulled directly from **Fig 1**. The dendrogram at the top of the heatmap was generated using pvclust. The red values are approximately unbiased (au) and the green are the bootstrap support (bp) values, both of which are generated by pvclust. The values shown are for illustrative purposes with the full dendrogram available in **S2 Fig**. The histogram at the bottom shows the distribution of all the z-score values in the heatmap. The heatmap uses a z-score normalization of log_2_(TPM) values for 39 genes of interest, pulled directly from **Fig 1**. The legend at the top is broken into three sections: project color, if the sample was drug treated, and sample life stage. The left hand legend is broken into two sections: the outer section denotes the WGCNA cluster and the inner section denotes if cluster matches the main or inverse WGCNA cluster expression pattern. On the right there is the gene name followed by the common name found in the source data file. Samples were labeled with first author, title, and bioproject from [18–29].

Gene expression was plotted for a previously described comprehensive list of genes for potential drug targets [56] (**Fig 5**). These genes exhibited life-cycle specific expression, with a large portion of these genes having increased expression in early stages of development such as the L3 and microfilariae stages (**Fig 5**). The genes that are upregulated in these early life stages include paramyosin, myosin heavy chain (MHC), cystatin, glutathione S-transferase, troponin, calreticulin, small heat shock protein, venom allergen-like protein, and putative major antigen. These genes are important for development of the nematode.

## Discussion

### Benefits of a unified analysis

A unified meta-analysis highlights similarities and variation between independently published studies. Such analyses can provide validation of prior results as well as facilitate new comparisons outside the scope of the original studies. This study shows that largely the transcriptome of the worms are similar across all studies discussed. There is consistent gene expression for adult males and microfilariae across several studies spanning several continents and a decade of research. This meta-analysis also revealed a corresponding lack of variation between treated and untreated samples by increasing the number of untreated samples available for comparison. It also highlighted differences between the adult female worms in different studies from the same time frame, which was only possible through a direct comparison in the unified meta-analysis (**S4, S5 and S6 Figs**). There would be benefits to developing a web resource to enable users to select their own comparisons (e.g. if a user wants to only compare all adult female profiles to the response to doxycycline treatment) within this unified analysis and to facilitate the analysis of data in the same framework. While that is beyond the scope of this work, we have provided a single table that includes the z-scores from the unified analysis for all genes for all conditions that includes the annotation, GO terms, and Interpro terms (**S5 Table**). Transcriptome sequencing of worms in future studies and their analysis in this framework could facilitate the understanding of differences between worms being used in studies, like the differences observed between adult female worms in these transcriptomics studies.

### Current landscape of *B*. *malayi* transcriptomics data

Differential expression tools have evolved over time such that we did not expect to fully recapitulate prior results with current methods. Life cycle specific gene expression remained consistent across several of the reanalyzed studies [18,22,24,26] spanning a decade of research and was a uniting factor in this meta-analysis. This is remarkable given the technical developments that have happened across the entire transcriptomics process from RNA isolation and library construction to Illumina sequencing platforms and differential expression analysis tools. While one might anticipate that there would be batch effects associated with laboratories, shipping conditions, media, and processing, the transcriptional pattern for males and microfilariae overall remained largely consistent across all studies. This may be excellent news for the community since we can then expect that the results between laboratories are consistent and reproducible. However, this contrasts with prior observations that suggested isolating RNA after freezing or culturing worms affects the transcriptional profile [20]. In addition, this is not what we observed for the two groups of adult female worms. We were unable to identify a specific reason why there are two expression profiles for adult females, despite examining RNA isolation method, library preparation technique, shipping method, mitochondrial expression, and potential sample artifacts. One possibility is that there is an arrest in reproduction and the contribution of developing offspring is missing in these worms. These differences in the adult females are so extensive, including more than 7,000 differentially expressed genes, it suggests that the worms may respond differently in experiments (**S5 Fig**). Culture acclimation, transit time, and transit conditions were not reported in the original studies, which could impact the transcriptional profile of the nematodes and inform interpretation of experimental results. Standards for reporting this information may be needed. Given the decreasing costs of transcriptome sequencing, it may be advisable in the future for all studies to capture transcriptomic profiles of worms during experiments, even those that would not normally focus on differential expression, like drug treatment studies. These transcriptomic profiles can then be used to benchmark the state of the worms in the study and improve experimental comparisons.

### Reproducibility of original study designs

We were able to replicate many of the results of the original studies included in this meta-analysis [18,20,22,24–29] either through the unified analysis or with more targeted analyses. But our reanalysis of prior individual studies yielded different conclusions for the studies examining *B*. *malayi* gene expression during *in vitro* or *in vivo* drug treatment [19,23]. While the previous analysis using an exact test identified genes detected as differentially expressed upon drug treatment, largely our re-analysis of the individual projects with the log linear model reveals no statistically significant differentially expressed genes upon *in vitro* or *in vivo* drug treatment relative to the controls (**S1 File**). This may not be surprising given that the doses of drugs given *in vivo* were not enough to reduce the worm burdens for each life stage [23]. In addition, these drugs generally may not be effective *in vivo* in gerbils. For example, ivermectin has been reported to be ineffective when used in gerbils [57]. In one study, DEC was ineffective in gerbils up to 300 mg/kg DEC administered for 5 days [58], while other studies show that it is effective and targets adult worms [59]. The effect of albendazole in the treatment of lymphatic filariasis is unclear in humans and gerbils [60, 61]. In the pairwise comparison between treated and untreated microfilariae [28], the number of originally reported differentially expressed genes (1,079) and the number in our reanalysis (1,423) are considered comparable, particularly given that different versions of the genome and thus different annotations were used. to interrogate this further.

### Updates to genome annotation

During this meta-analysis, we identified a number of regions annotated with genes that adversely affected normalization and as such the subsequent statistical tests and analyses. These annotated genes are open reading frames that overlap highly expressed non-coding RNAs, like the rRNAs. As such they contain a disproportionate number of reads, which affects the normalization in the differential expression analysis. We have provided an updated genome annotation file for *B*. *malayi* (**S2 File**) based off the WormBase WS276 version where the genes that overlap with predicted rRNAs have been removed. In addition, we identified poorly supported genes that were not expressed in any study. We cannot rule out that they are just not expressed in other conditions, although they were atypical in their placement on small unplaced contigs, and the majority had 1–2 exons. We have marked these genes with a note in this updated gff file, as described above, such that they can be examined in future genome annotation efforts.

### Data sharing and data re-use

All data used in this meta-analysis was downloaded from the SRA (**Table 1**). The continuous addition of new RNA-sequence data into the SRA allows future projects to easily build off and include existing data. The ability to download and analyze the data from the SRA allows researchers to answer research questions without having to invest in generating the data themselves. In addition, deposition of sequence data enables and facilitates the reanalysis of the original study with more recent and updated algorithms that did not exist at the time of the original study. Reanalyses are important to ensure that the results of the study are consistent, reliable, and not limited by the existing statistical tests and methods of the time. Lastly, data from other studies can extend existing studies and increase the number of replicates enabling more rigorous statistical testing for significance. In this case, the meta-analysis demonstrates the reproducibility of studies from numerous different research groups using different sequence technologies/library preps. Ensuring consistent results provides confidence in the data published and reliability in the technologies and statistical methods used. However, data re-use and re-analysis of prior studies is not without its challenges. The lack of some experimental details, details about algorithm version, details about algorithm options, and the availability of in-house code are all barriers to reproducing analyses with fidelity.

Overall, this meta-analysis shows the power of data sharing and re-use, providing insight into the current transcriptomic landscape and a unified pipeline that other researchers can use in the future.

## Supporting information

S1 File Summary of Papers and Individual Re-analysis of Studies in Word. (DOCX)

S2 File Updated GFF file with Genes Overlapping with Predicted rRNA’s Removed. (ZIP)

S3 File WGCNA Cluster Composition in Excel. (XLSX)

S4 File Functional Term Enrichment for WGCNA Clusters in Excel. (XLSX)

S1 Fig Schematic of the Removed Genes that Overlapped Predicted rRNAs. An adapted schematic of annotated *B*. *malayi* genes overlapping predicted rRNA features from IGV. The blue boxes denote exons with white arrows showing the respective strand. All gene locations and sizes are not to scale.
(PDF)

S2 Fig High Resolution Image of Heat Map in Fig 1. An adapted version of **Fig 1** with the sample names at the top of each column without the dendrogram. The sample names are from the dendrogram presented in **S3 Fig**. The histogram at the bottom shows the distribution of all the z-score values in the heatmap. The heatmap uses a z-score normalization of log_2_(TPM) values for 9,727 differentially expressed genes between 237 samples reanalyzed from 12 projects. The legend at the top is broken into three sections: project color, if the sample was drug treated, and sample life stage. The left hand legend is broken into two sections: the outer section denotes the WGCNA cluster and the inner section denotes if cluster matches the main or inverse WGCNA cluster expression pattern. Samples were labeled with first author, title, and bioproject from [18–29].
(PDF)

S3 Fig High Resolution Image of Pvclust Dendrogram in Fig 1 with Sample Labels and Support Values. The pvclust dendrogram, provided in **Fig 1**, with all sample names and support values shown. The red values are approximately unbiased (au) and the green are the bootstrap support (bp) values. The size of support values above a height of 0.6 have been increased for illustrative purposes. The sample names are included in the dendrogram.
(PDF)

S4 Fig Differential Expression of 18 Male Samples, 18 mf Samples, 9 Female Samples in Group 1, and 9 Female Samples in Group 2. The dendrogram at the top of the heatmap was generated using pvclust. The red values are approximately unbiased (au) and the green are the bootstrap support (bp) values, both of which are generated by pvclust. The values shown are for illustrative purposes. The samples included are select adult male samples, microfilariae, and from the two groups of adult females. The heatmap uses a z-score normalization of log_2_(TPM) values for the 8,697 differentially expressed genes. The legend at the top is broken into three sections: project color, if the sample was drug treated, and sample life stage. The left hand legend is broken into two sections: the outer section denotes the WGCNA cluster and the inner section denotes if cluster matches the main or inverse WGCNA cluster expression pattern. Samples were labeled with first author, title, and bioproject from [19,20,23].
(PDF)

S5 Fig Differential Expression between Adult Female Samples. The dendrogram at the top of the heatmap was generated using pvclust. The red values are approximately unbiased (au) and the green are the bootstrap support (bp) values, both of which are generated by pvclust. The values shown are for illustrative purposes. The samples included are select adult female samples. The heatmap uses a z-score normalization of log_2_(TPM) values for the 7,785 differentially expressed genes. The legend at the top is broken into three sections: project color, if the sample was drug treated, and sample life stage. The left hand legend is broken into two sections: the outer section denotes the WGCNA cluster and the inner section denotes if cluster matches the main or inverse WGCNA cluster expression pattern. Samples labeled with first author, title, and bioproject from [19,20,23–25]
(PDF)

S6 Fig Differential Expression of Adult Female Samples Based on Sample Culture Condition. The dendrogram at the top of the heatmap was generated using pvclust. The red values are approximately unbiased (au) and the green are the bootstrap support (bp) values, both of which are generated by pvclust. The values shown are for illustrative purposes. The samples included are select adult female samples. The heatmap uses a z-score normalization of log_2_(TPM) values for the 2,906 differentially expressed genes. The legend at the top is broken into four sections: project color, if the sample was drug treated, sample life stage, and media status. The left hand legend is broken into two sections: the outer section denotes the WGCNA cluster and the inner section denotes if cluster matches the main or inverse WGCNA cluster expression pattern. Samples were labeled with first author, title, and bioproject from [18–20,22–25].
(PDF)

S1 Table Sequence Read Archive Accessions for 244 Data Sets Analyzed and a Compilation of Metadata and Properties. (XLSX)

S2 Table Genes Removed from the Analysis due to the Application of the CPM Filter. (XLSX)

S3 Table Differentially Expressed Genes in Soma and Germline Re-analysis. (XLSX)

S4 Table Differentially Expressed Genes Following Tetracycline Treatment. (XLSX)

S5 Table Z-scores, Annotation, GO Terms, and Interpro Terms for All Genes and All Experiments in the Unified Meta-analysis. (XLSX)
