# Supplementary material for: Updated annotation and meta-analysis of Brugia malayi transcriptomics data reveals consistent transcriptional profiles across time and space with some study-specific differences in adult female worm transcriptional profiles
Source: PLoS Negl Trop Dis. 2024 Sep 26;18(9):e0012511. doi: 10.1371/journal.pntd.0012511 (PMC11460672; doi:10.1371/journal.pntd.0012511)
Supplement: S1 File — (DOCX) [file pntd.0012511.s001.docx]

# SUPPLEMENTAL FILE 1: SUMMARY OF PAPERS

## CHOI ET AL 2011 [1]: A DEEP SEQUENCING APPROACH TO COMPARATIVELY ANALYZE THE TRANSCRIPTOME OF LIFECYCLE STAGES OF THE FILARIAL WORM, *BRUGIA MALAYI*

In 2011, the first *B. malayi* transcriptomics study (PRJEB2709) was published identifying discrete transcriptional patterns across the life cycle [1]. Illumina GA IIx RNA-sequencing data (2x54-bp reads) was generated for adult females, adult males, eggs and embryos, immature microfilariae, mature microfilariae, L3 larvae, and L4 larvae. The reads were mapped to *B. malayi* reference genome using TopHat v1.0.14 [1, 2], filtered on mapping quality, counts with HTSeq v0.4.7 [3] with union mode using exon features aggregating on gene model. Counts were converted to RPKM and differentially expressed genes were identified using edgeR [4, 5] and a negative binomial model (*p*-value < 0.01). There were 2,430 differentially expressed genes identified in the original analysis with different transcriptome profiles identified between the life stage samples with more overlap in expression between adult females, embryos, microfilariae stages, and the larvae stage with many adult male-specific genes.


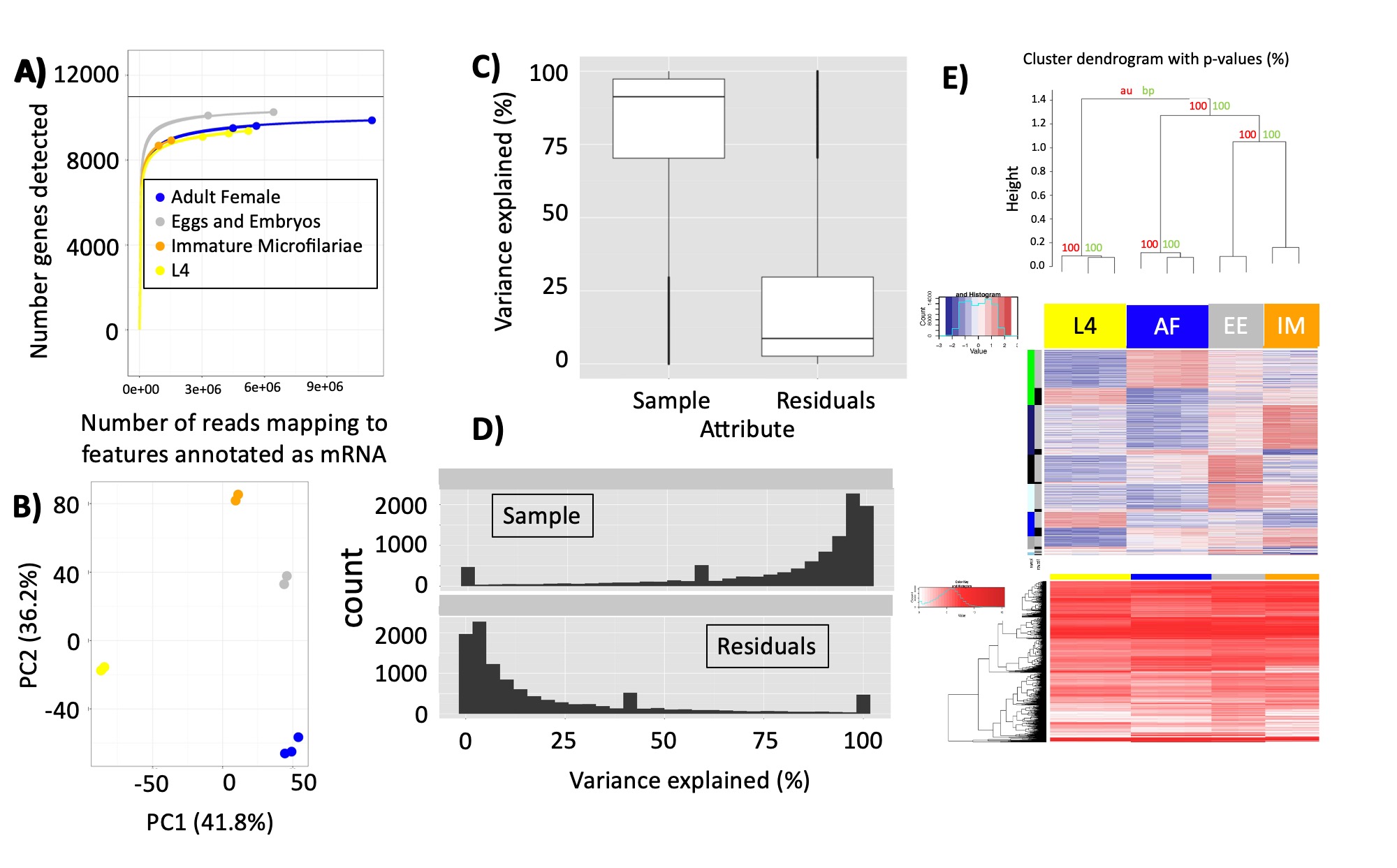


**Note: The heatmaps and PCA were generated using differentially expressed genes**

### Supplementary Figure 1: Reanalysis of lifecycle differential expression data reveals 8,050 differentially expressed genes. A) A rarefaction curve of the adult female, eggs and embryos, immature microfilariae, and L4 samples showing that these samples reach acceptable levels of saturation. B). These samples separate out based on life stage in a principal components analysis. The PCA plot was generated using z-score normalized log_2_(TPM) values of the differentially expressed genes C) Linear mixed model assessing sample variation using varianceParition D) Histogram of the linear mixed model results, showing how many genes are responsible for a certain percentage of variation E) Differential expression results using the z-score normalized log_2_(TPM) values of the differentially expressed genes in the top plot and the log_2_(TPM) values of the differentially expressed genes in the bottom plot. Both plots are ordered based on the Pvclust dendrogram, which was generated using the z-score normalized log_2_(TPM) values of the differentially expressed genes. The top annotation bar denotes the sample life stage. In the top plot, the outer left annotation bar denotes the WGCNA cluster and the inner left annotation bar denotes whether the cluster matches the main expression profile (grey) or inverse expression profile (black).

## BALLESTEROS ET AL 2016 [6]: THE EFFECTS OF IVERMECTIN ON *BRUGIA MALAYI* FEMALES *IN VITRO*: A TRANSCRIPTOMIC APPROACH

Five years later, in 2016, three differential expression analyses were published. In one paper, the transcriptional response of worms from intraperitoneally infected male gerbils to ivermectin was measured (a) across time at the same dose and (b) at a single time point with varying doses [6]. The RNA-Seq reads were mapped to the *B. malayi* reference genome using TopHat [2], summary statistics calculated using picard [7], and counted with HTSeq with union mode using gene feature [6]. EdgeR [4, 5] was used to normalize read counts with the TMM method, estimate gene-specific dispersion values with the empirical Bayes method, estimate dispersion after fitting a negative binomial, and identify differential expression with the exact test with significance set at a FDR < 0.2 after Benjamani-Hochberg correction [6]. In this first part of the study (Bioproject PRJNA303987), worms were exposed to 100 nM ivermectin over time, reporting 34 differentially expressed genes after 24 h of exposure, 421 differentially expressed genes after 48 h, and 15 differentially expressed genes after 72 h group [6]. The second part of the study (Bioproject PRJNA303986) measured differential expression between 300 nM and 1 µM ivermectin to time matched controls at 48 h and 120 h. Across the four pairwise comparison, they identified 68-271 differentially expressed genes using the exact test while with the negative binomial generalized log-linear model we used, no differentially expressed genes were identified in any of these pairwise comparisons

###
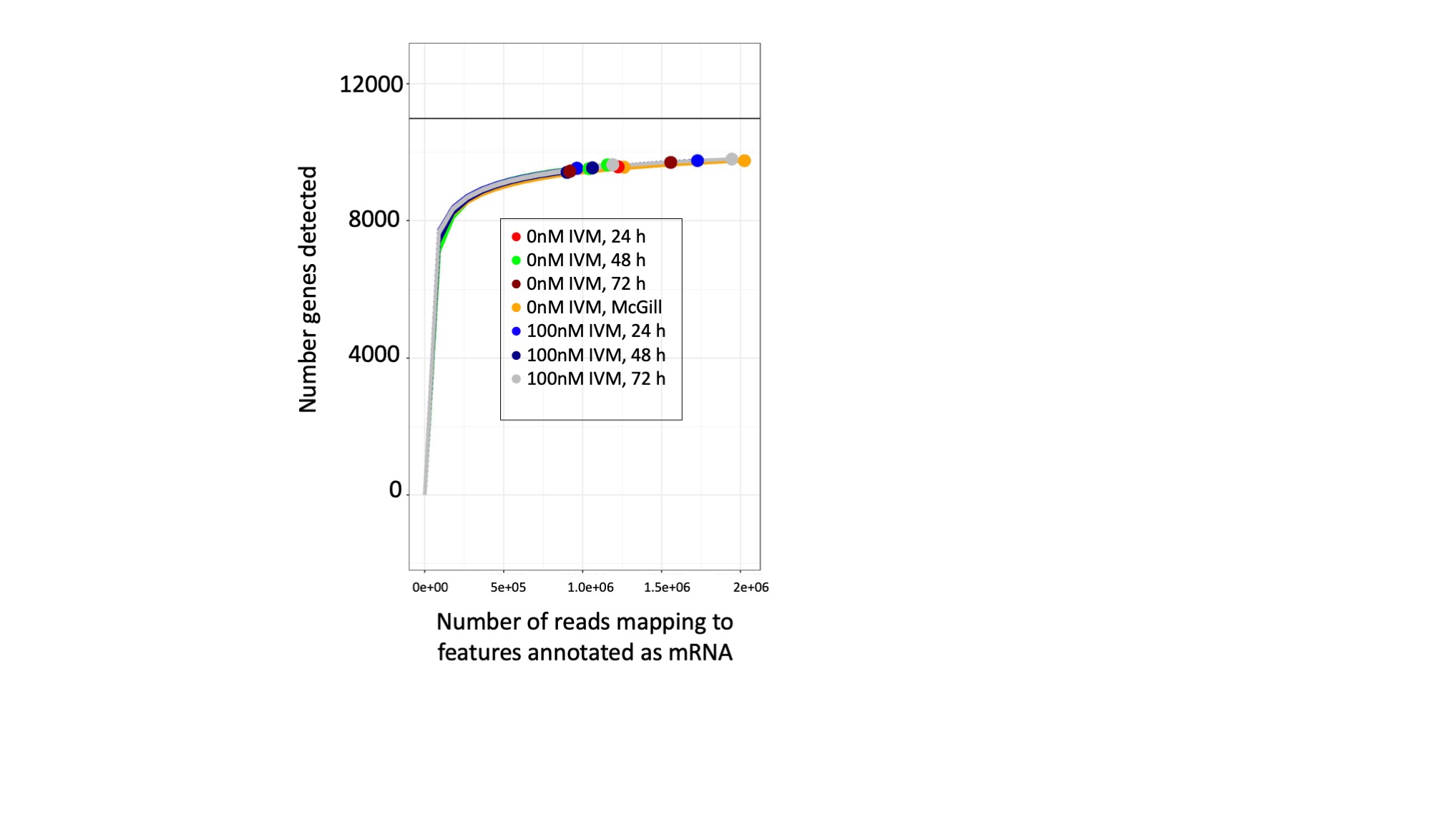
Study I Bioproject PRJNA303987

#### Supplemental Figure 2: Rarefaction curve of the samples colored by Ivermectin dose and exposure time. All samples in this study reached acceptable levels of saturation.


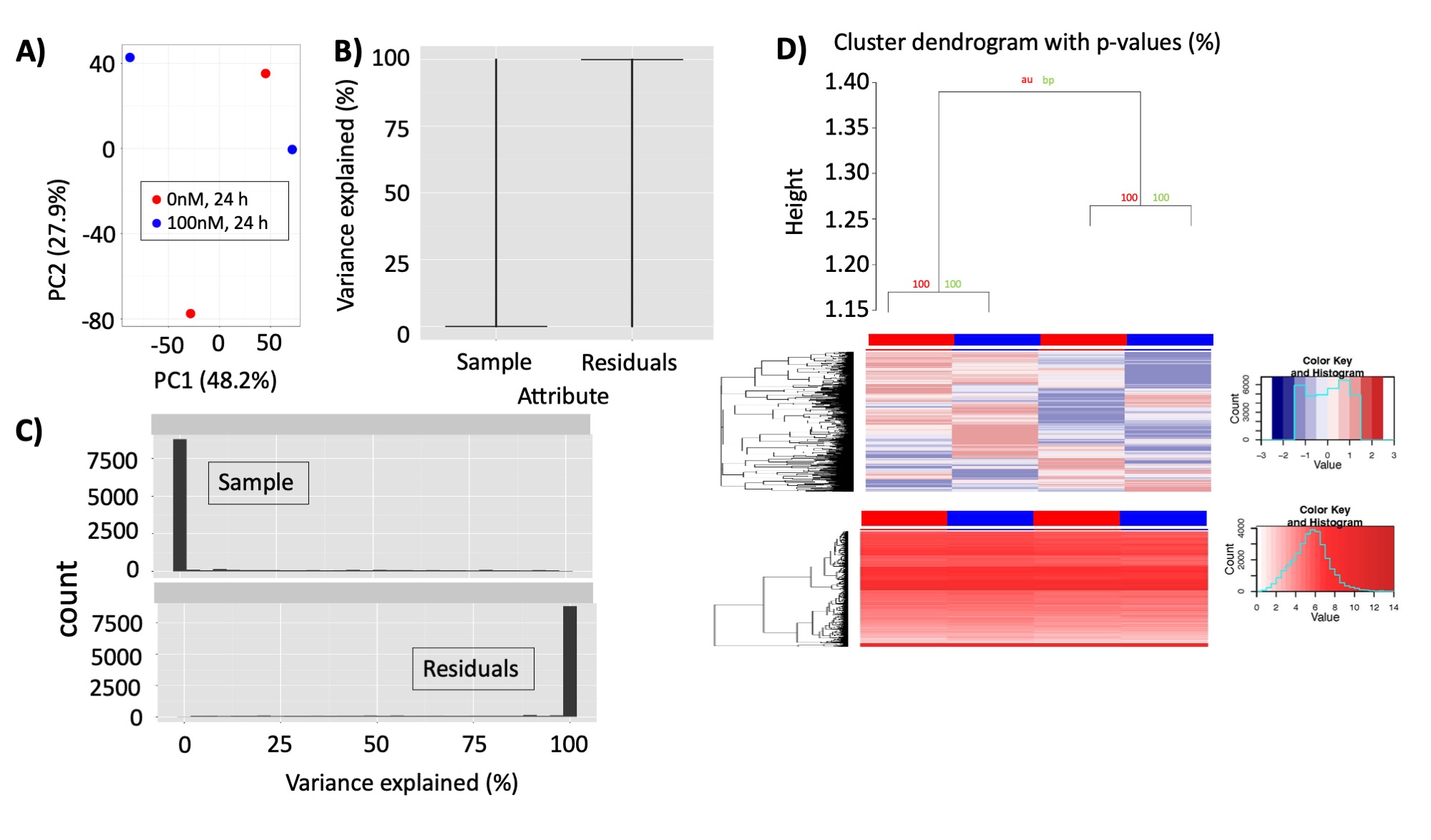


**Note: The heatmaps and PCA were generated using genes that passed the edgeR CPM filter**

#### Supplemental Figure 3: There is a lack of differential expression based on IVM treatment after 24 hours. A) The samples do not separate based on IVM treatment in a principal components analysis. The PCA plot was generated using z-score normalized log_2_(TPM) values of the genes passing the CPM filter B) Linear mixed model assessing sample variation using varianceParition C) Histogram of the linear mixed model results, showing how many genes are responsible for a certain percentage of variation D) A heatmap using the z-score normalized log_2_(TPM) values of the genes passing the CPM filter in the top plot and the log_2_(TPM) values of the genes passing the CPM filter in the bottom plot. Both plots are ordered based on the Pvclust dendrogram, which was generated using the z-score normalized log_2_(TPM) values of the genes passing the CPM filter. The top annotation bar denotes the ivermectin dose


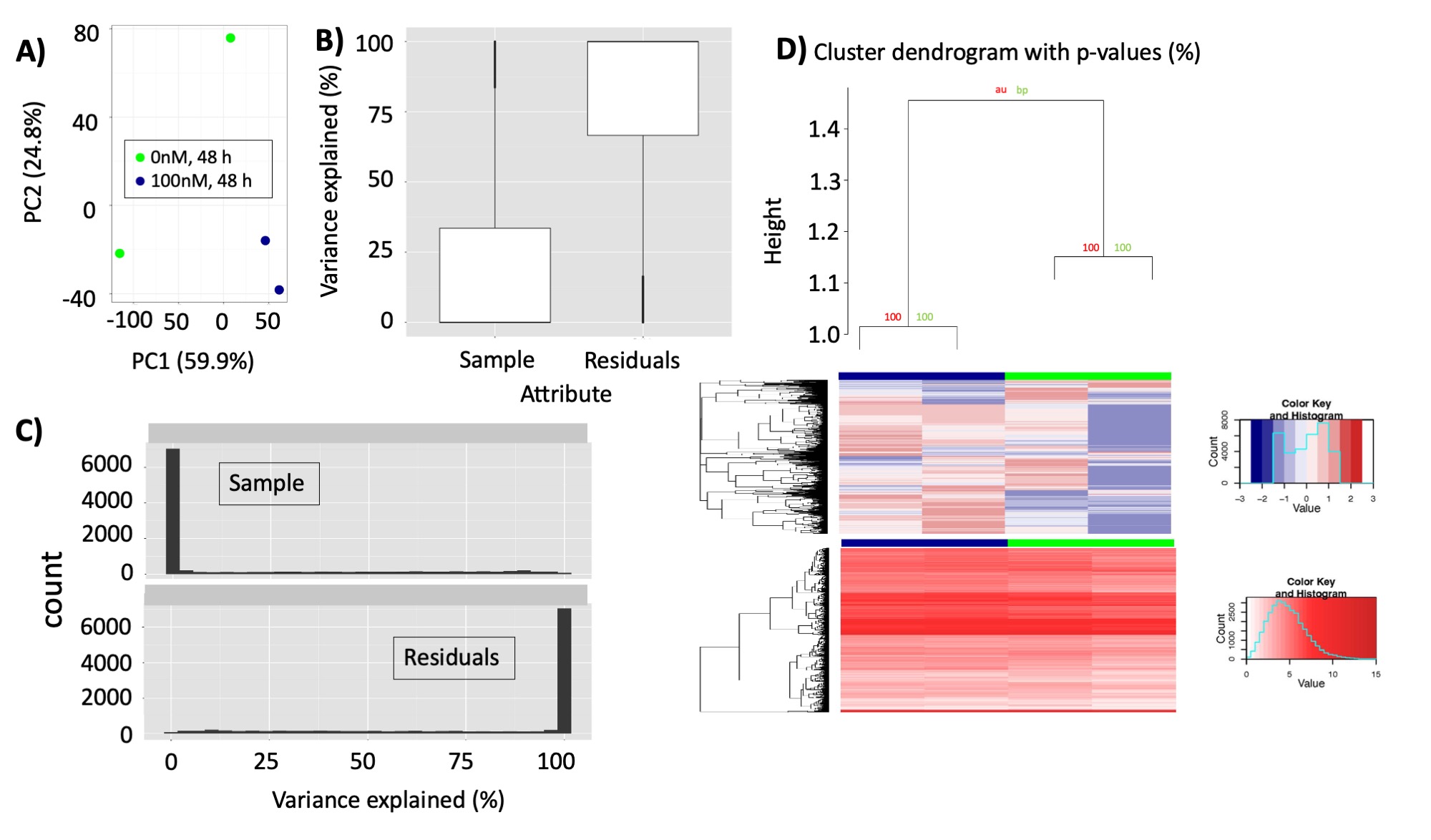


**Note: The heatmaps and PCA were generated using genes that passed the edgeR CPM filter**

#### Supplemental Figure 4: There is a lack of differential expression based on IVM treatment after 48 hours. A) The samples do not separate based on IVM treatment in a principal components analysis. The PCA plot was generated using z-score normalized log_2_(TPM) values of the genes passing the CPM filter B) Linear mixed model assessing sample variation using varianceParition C) Histogram of the linear mixed model results, showing how many genes are responsible for a certain percentage of variation D) A heatmap using the z-score normalized log_2_(TPM) values of the genes passing the CPM filter in the top plot and the log_2_(TPM) values of the genes passing the CPM filter in the bottom plot. Both plots are ordered based on the Pvclust dendrogram, which was generated using the z-score normalized log_2_(TPM) values of the genes passing the CPM filter. The top annotation bar denotes the ivermectin dose


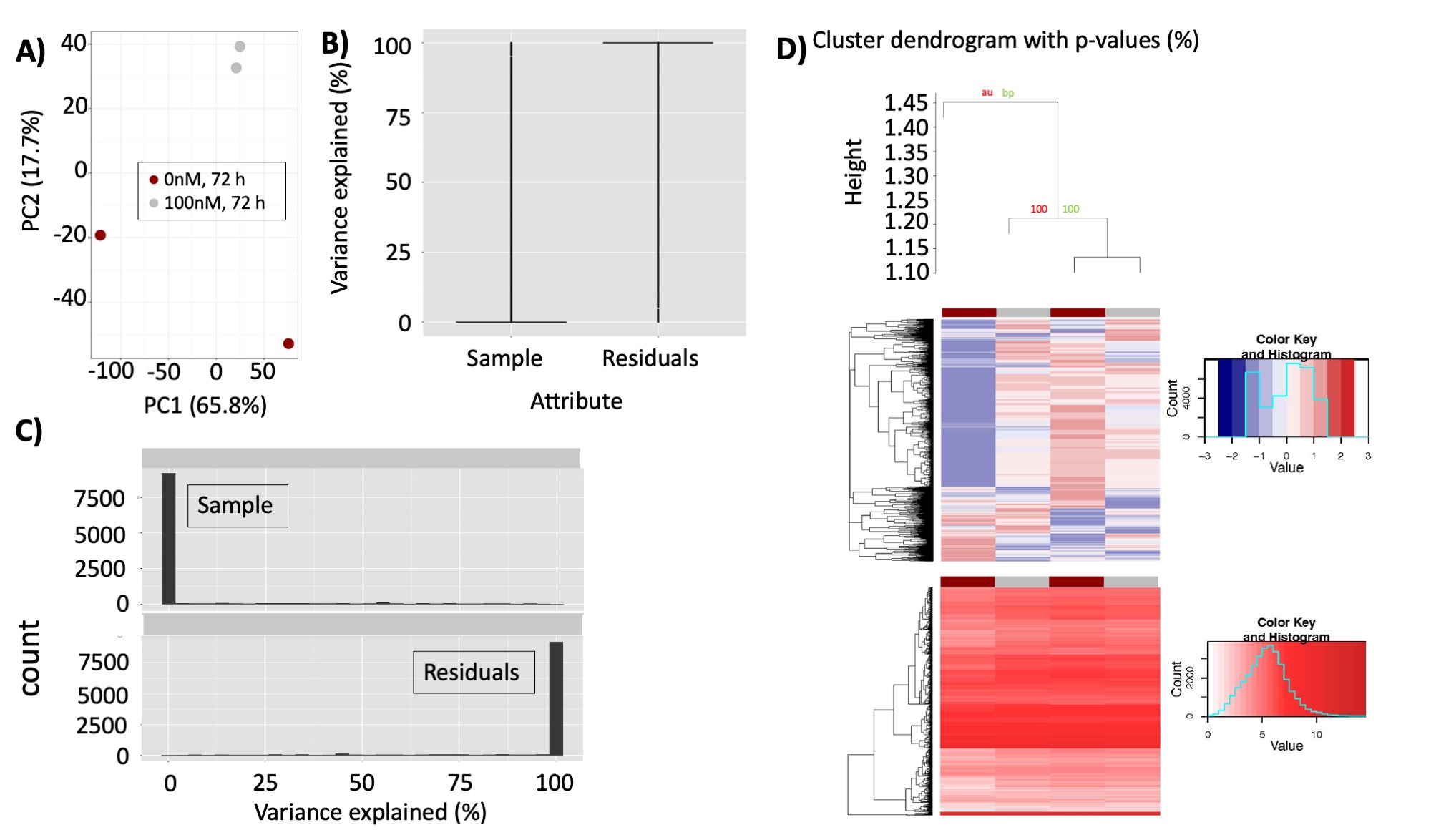


**Note: The heatmaps and PCA were generated using genes that passed the edgeR CPM filter**

#### Supplemental Figure 5: There is a lack of differential expression based on IVM treatment after 72 hours. A) The samples do not separate based on IVM treatment in a principal components analysis. The PCA plot was generated using z-score normalized log_2_(TPM) values of the genes passing the CPM filter B) Linear mixed model assessing sample variation using varianceParition C) Histogram of the linear mixed model results, showing how many genes are responsible for a certain percentage of variation D) A heatmap using the z-score normalized log_2_(TPM) values of the genes passing the CPM filter in the top plot and the log_2_(TPM) values of the genes passing the CPM filter in the bottom plot. Both plots are ordered based on the Pvclust dendrogram, which was generated using the z-score normalized log_2_(TPM) values of the genes passing the CPM filter. The top annotation bar denotes the ivermectin dose

###
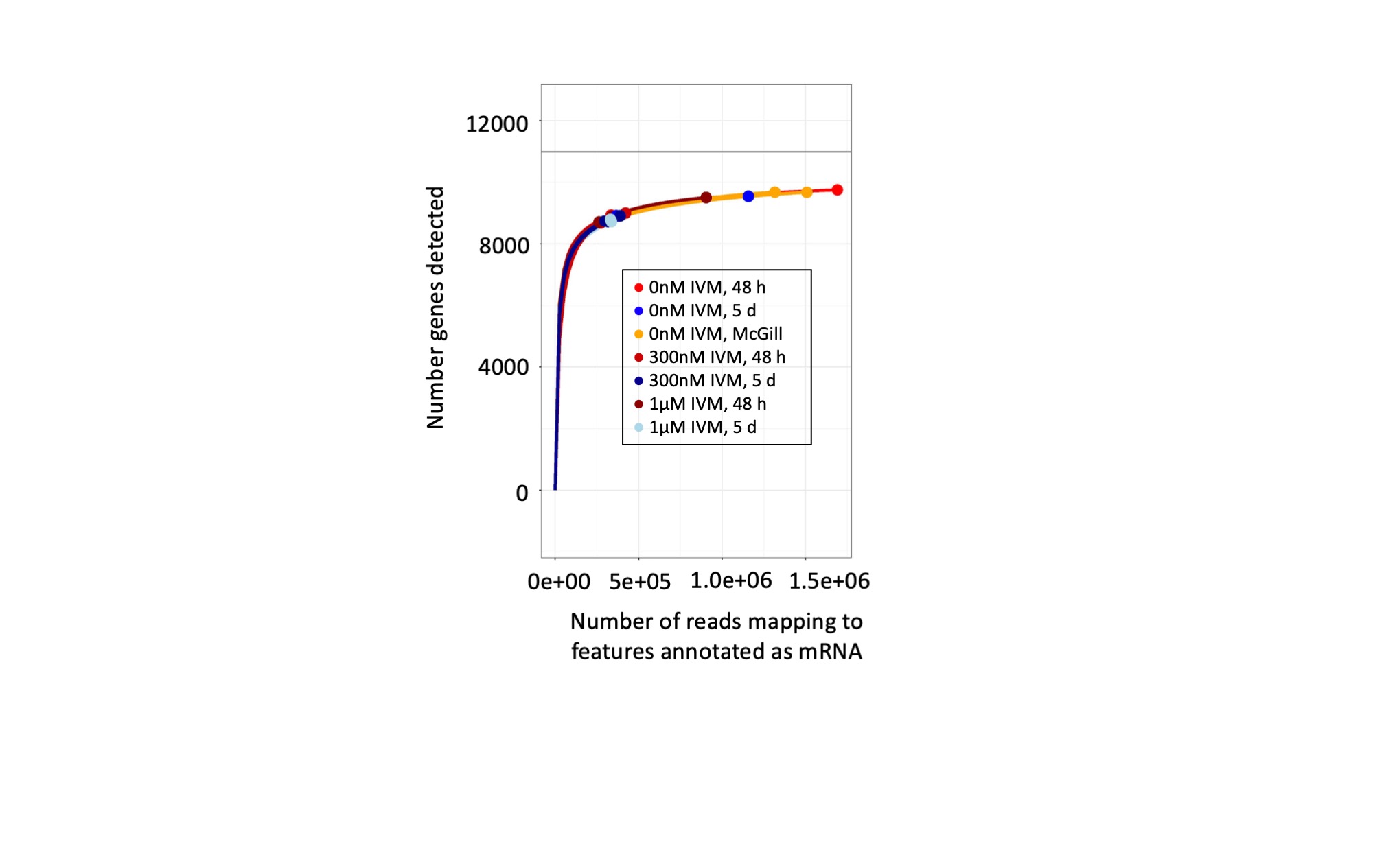
Study II Bioproject PRJNA303986

#### Supplemental Figure 6: Rarefaction curve of the samples used in study II. All samples analyzed in this study reach acceptable levels of saturation


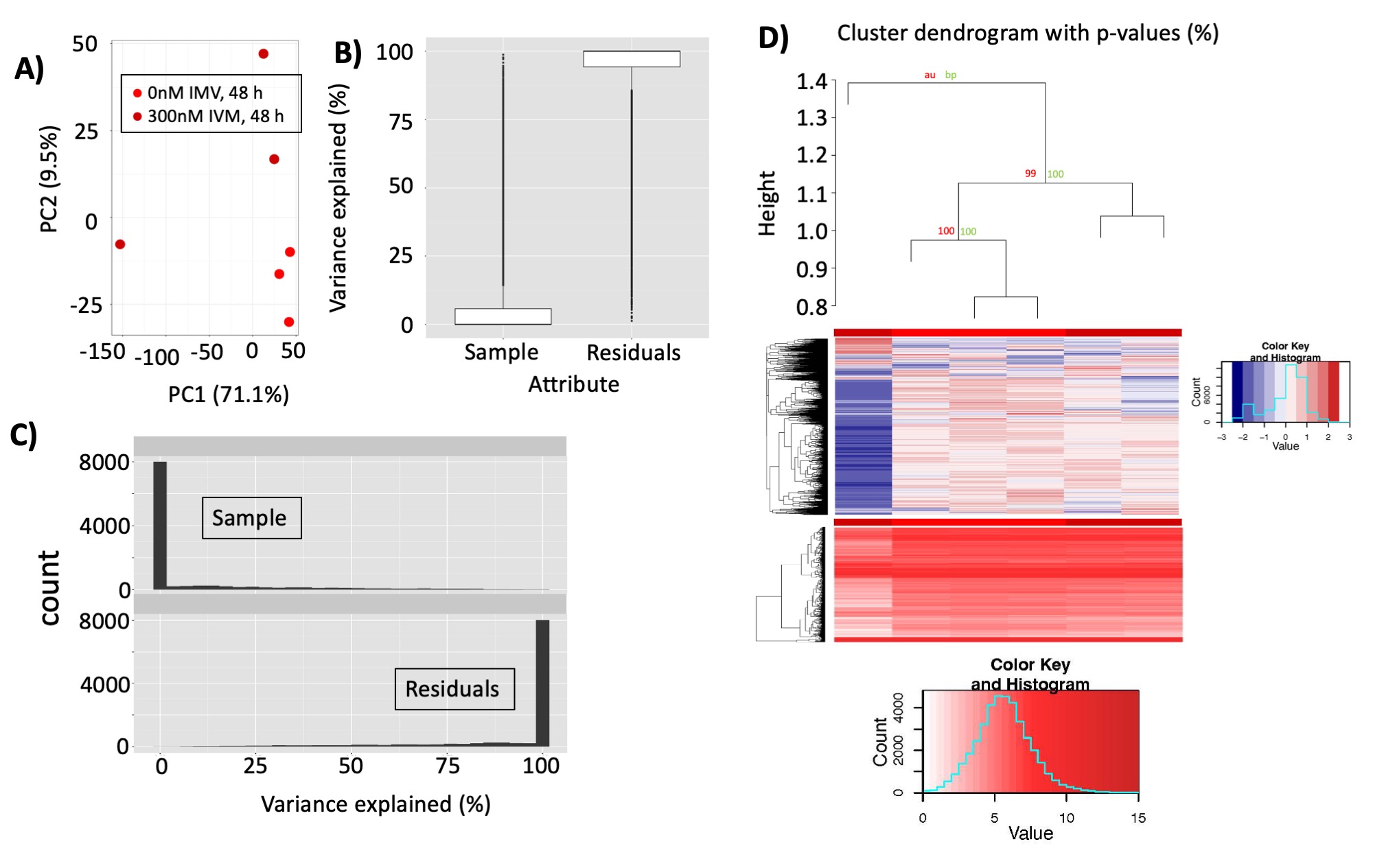


**Note: The heatmaps and PCA were generated using genes that passed the edgeR CPM filter**

#### Supplemental Figure 7: There is a lack of differential expression based on 300nM IVM treatment after 48 hours. A) The samples do not separate based on IVM treatment in a principal components analysis. The PCA plot was generated using z-score normalized log_2_(TPM) values of the genes passing the CPM filter B) Linear mixed model assessing sample variation using varianceParition C) Histogram of the linear mixed model results, showing how many genes are responsible for a certain percentage of variation D) A heatmap using the z-score normalized log_2_(TPM) values of the genes passing the CPM filter in the top plot and the log_2_(TPM) values of the genes passing the CPM filter in the bottom plot. Both plots are ordered based on the Pvclust dendrogram, which was generated using the z-score normalized log_2_(TPM) values of the genes passing the CPM filter. The top annotation bar denotes the ivermectin dose.


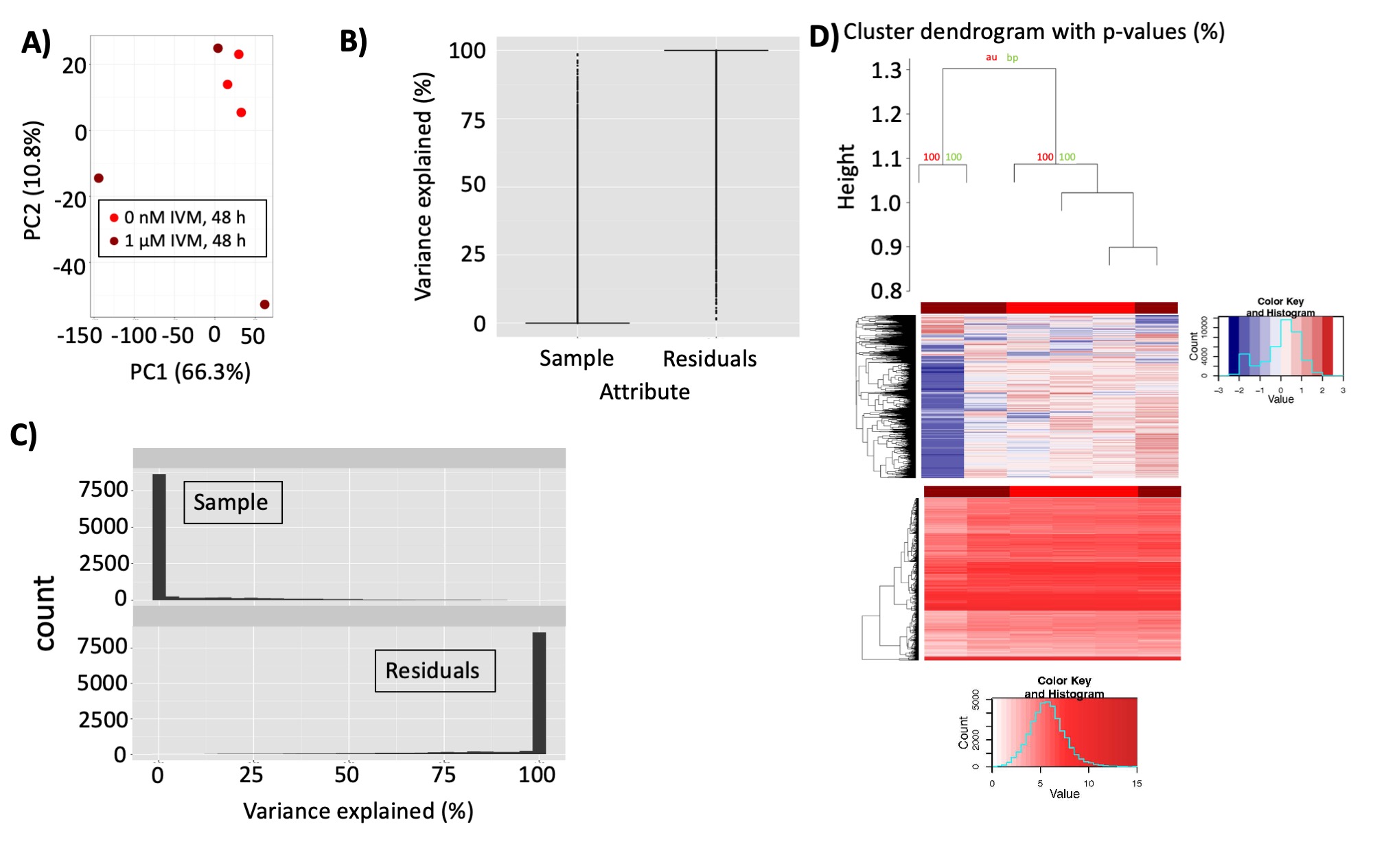


**Note: The heatmaps and PCA were generated using genes that passed the edgeR CPM filter**

#### Supplemental Figure 8: There is a lack of differential expression based on 1µM IVM treatment after 48 hours. A) The samples do not separate based on IVM treatment in a principal components analysis. The PCA plot was generated using z-score normalized log_2_(TPM) values of the genes passing the CPM filter B) Linear mixed model assessing sample variation using varianceParition C) Histogram of the linear mixed model results, showing how many genes are responsible for a certain percentage of variation D) A heatmap using the z-score normalized log_2_(TPM) values of the genes passing the CPM filter in the top plot and the log_2_(TPM) values of the genes passing the CPM filter in the bottom plot. Both plots are ordered based on the Pvclust dendrogram, which was generated using the z-score normalized log_2_(TPM) values of the genes passing the CPM filter. The top annotation bar denotes the ivermectin dose


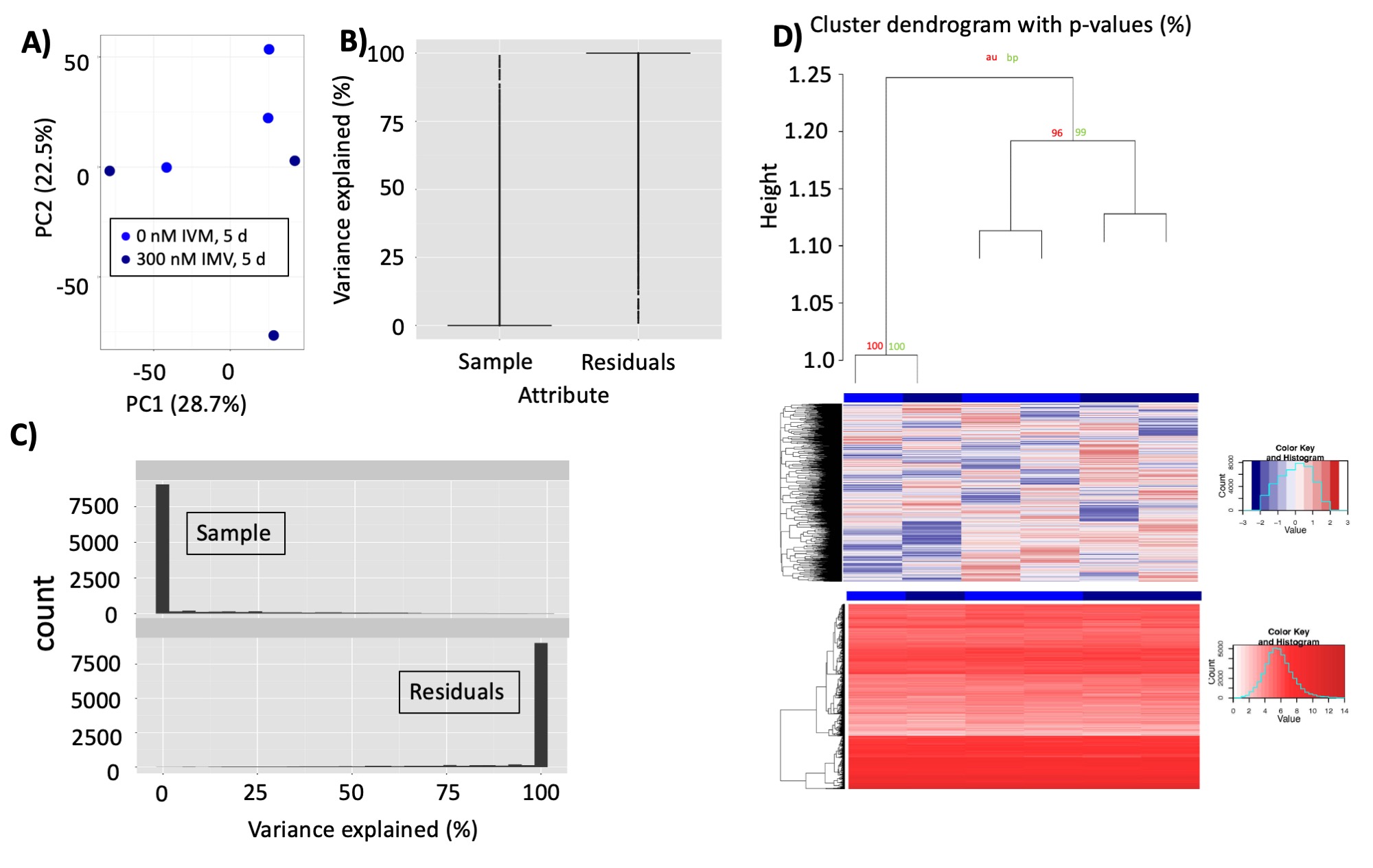


**Note: The heatmaps and PCA were generated using genes that passed the edgeR CPM filter**

#### Supplemental Figure 9: There is a lack of differential expression based on 300 nM IVM treatment after 48 hours. A) The samples do not separate based on IVM treatment in a principal components analysis. The PCA plot was generated using z-score normalized log_2_(TPM) values of the genes passing the CPM filter B) Linear mixed model assessing sample variation using varianceParition C) Histogram of the linear mixed model results, showing how many genes are responsible for a certain percentage of variation D) A heatmap using the z-score normalized log_2_(TPM) values of the genes passing the CPM filter in the top plot and the log_2_(TPM) values of the genes passing the CPM filter in the bottom plot. Both plots are ordered based on the Pvclust dendrogram, which was generated using the z-score normalized log_2_(TPM) values of the genes passing the CPM filter. The top annotation bar denotes the ivermectin dose.


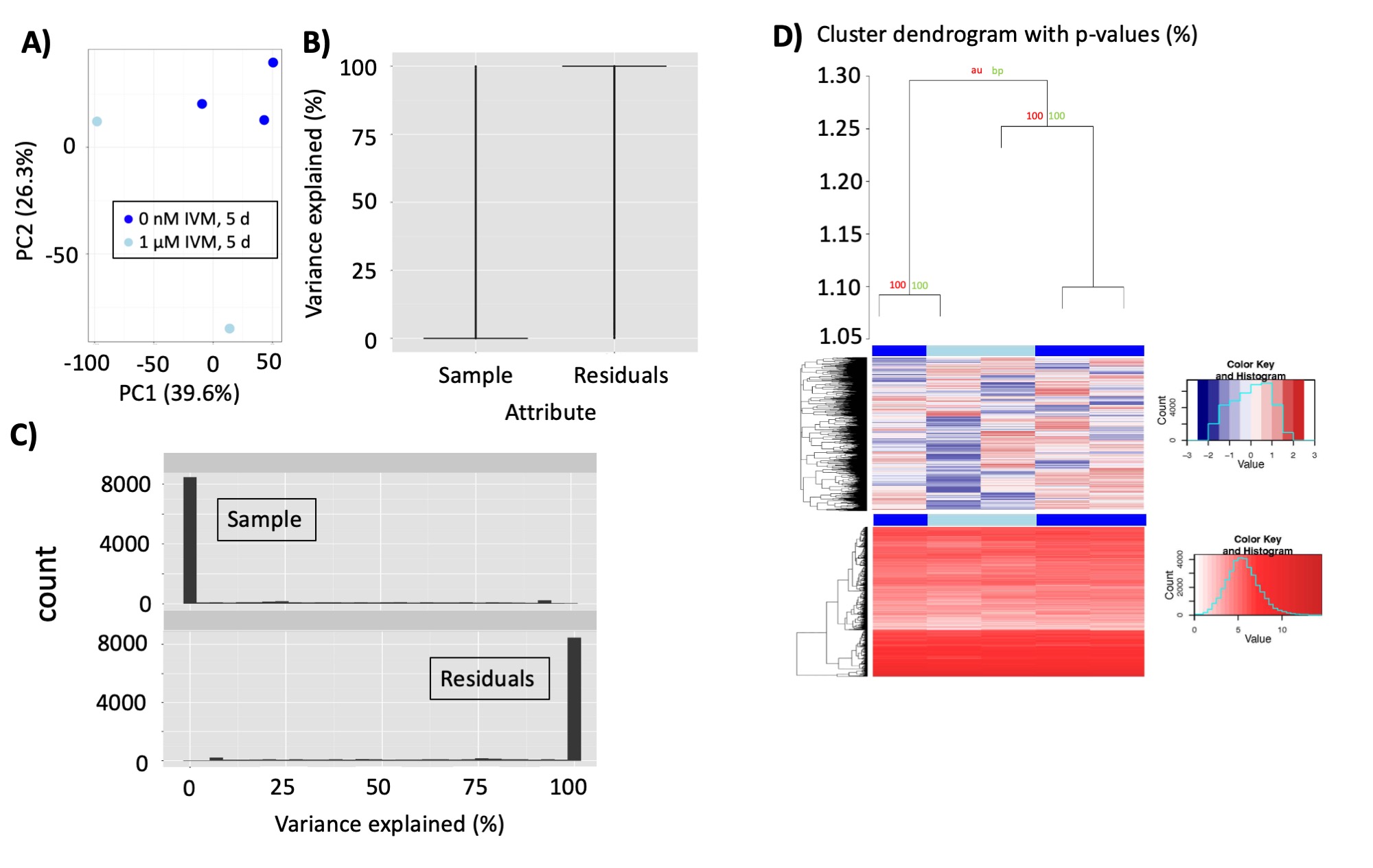


**Note: The heatmaps and PCA were generated using genes that passed the edgeR CPM filter**

#### Supplemental Figure 10: There is a lack of differential expression based on 1µM IVM treatment after 5 days. A) The samples do not separate based on IVM treatment in a principal components analysis. The PCA plot was generated using z-score normalized log_2_(TPM) values of the genes passing the CPM filter B) Linear mixed model assessing sample variation using varianceParition C) Histogram of the linear mixed model results, showing how many genes are responsible for a certain percentage of variation D) A heatmap using the z-score normalized log_2_(TPM) values of the genes passing the CPM filter in the top plot and the log_2_(TPM) values of the genes passing the CPM filter in the bottom plot. Both plots are ordered based on the Pvclust dendrogram, which was generated using the z-score normalized log_2_(TPM) values of the genes passing the CPM filter. The top annotation bar denotes the ivermectin dose.

## BALLESTEROS ET AL 2016 [8]: THE EFFECT OF *IN VITRO* CULTIVATION ON THE TRANSCRIPTOME OF ADULT *BRUGIA MALAYI*

An additional paper from the same group measured the effect *in vitro* cultivation has on the transcriptome profile, comparing freshly isolated worms, upon arrival of worms, or after acclimating and subculturing for 2 days or 5 days [8]. This is an important and pragmatic study since many researchers do not maintain the complex life cycle, instead receiving worms through the post from the FR3 resource center. Adult male gerbils were injected with L3s subcutaneously and adult female worms were isolated from the peritoneal cavity [8]. Differentially expressed genes were found across all the time points of cultivation, indicating that the time point when the worms are isolated affecting the transcriptomic profile of the nematode [8]. The 150 bp single end Illumina MiSeq reads (Bioproject PRJNA294426) were aligned to the *B. malayi* WS243 reference genome using TopHat2 v0.6 [2]. Read counts were generated on transcript features using HTSeq-Count v1.0.0 in union mode [3]. EdgeR v3.10.5 [4, 5] was used to normalize read counts with the TMM method, estimate gene-specific dispersion values with the empirical Bayes method, estimate dispersion after fitting a negative binomial, and identify differential expression in all possible pairwise comparisons with the exact test with significance set at an FDR < 0.01 after Benjamani-Hochberg correction. Between 491 and 2,169 genes were identified as differentially expressed across all the pairwise comparisons. Additional post-hoc filtering focused the analysis on 2-fold up- and down-regulated genes, which yielded 138-562 differentially expressed genes.


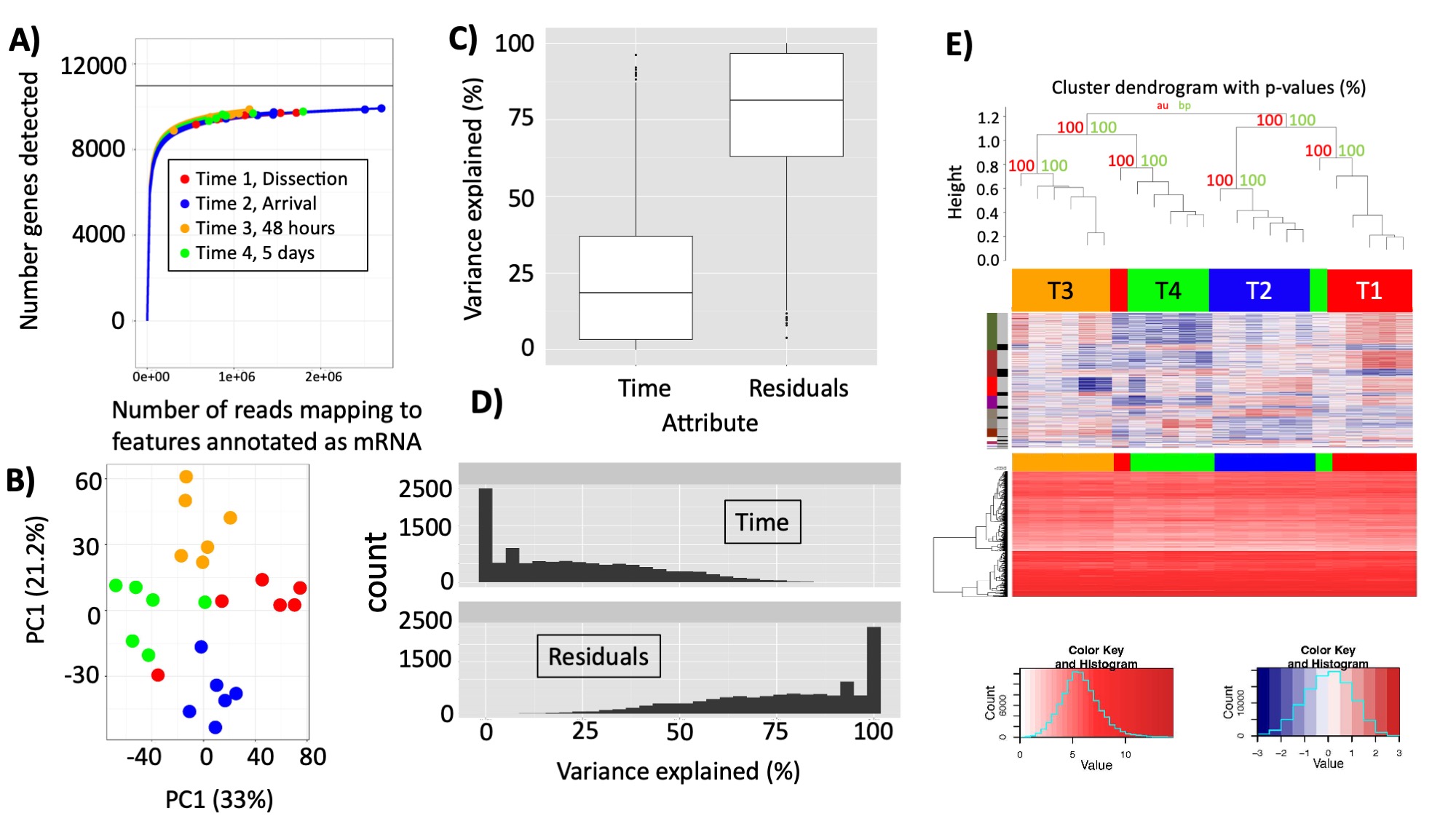


**Note: The heatmaps and PCA were generated using differentially expressed genes**

### Supplemental Figure 11: Differential expression reanalysis of RNA isolation data reveals 4,242 differentially expressed genes. A) A rarefaction curve of the four RNA isolation time points showing that these samples reach acceptable levels of saturation. B). Principal components analysis of samples that was generated using z-score normalized log_2_(TPM) values of the differentially expressed genes C) Linear mixed model assessing sample variation using varianceParition D) Histogram of the linear mixed model results, showing how many genes are responsible for a certain percentage of variation E) Differential expression results using the z-score normalized log_2_(TPM) values of the differentially expressed genes in the top plot and the log_2_(TPM) values of the differentially expressed genes in the bottom plot. Both plots are ordered based on the Pvclust dendrogram, which was generated using the z-score normalized log_2_(TPM) values of the differentially expressed genes. The top annotation bar denotes the RNA isolation time point. In the top plot, the outer left annotation bar denotes the WGCNA cluster and the inner left annotation bar denotes whether the cluster matches the main expression profile (grey) or inverse expression profile (black).

## LIBRO ET AL 2016 [9]: CHARACTERIZATION OF INNATE IMMUNITY GENES IN THE PARASITIC NEMATODE *BRUGIA MALAYI*

A study looking to characterise the innate immunity genes of *B. malayi* was published in 2016 [9]. In order to ascertain the pathways involved in *B. malayi* immunity, adult female nematodes were exposed to *Escherichia coli*, *Bacillus amyloliquefaciens*, double-stranded (ds) RNA, and dsDNA for two different timepoints, with two replicates collected per timepoint. Worms were also exposed to medium containing antibiotic (CplusA) and without antibiotic (CnoA), both for 24 and 36 hours. The antibiotic solution contained penicillin, streptomycin, and amphotericin. Bulk RNA-Sequencing of the exposed and unexposed worms was performed to assess the effect on *B. malayi* transcriptome. The cDNA was sequenced on an Illumina miSeq platform, producing 150bp paired end reads. Reads were mapped to the WS247 *B. malayi* reference genome using TopHat. Differential expression was calculated using pairwise comparisons between treated and untreated samples using Cuffdiff and statistical significance cutoff of FDR < 0.01. In their pairwise differential expression analysis, between identified differentially expressed genes in all of the conditions. This study was not reanalyzed due to missing samples in the SRA.

## GROTE ET AL 2017 [10]: DEFINING *BRUGIA MALAYI* AND *WOLBACHIA* SYMBIOSIS BY STAGE-SPECIFIC DUAL RNA-SEQ

In 2017, the first dual species transcriptomics analysis was completed, simultaneously analyzing the nematode transcriptome with its *Wolbachia* endosymbiont (*w*Bm) transcriptomes [10]. Our reanalysis will focus on the *B. malayi* expression data, since the *w*Bm data was reanalyzed previously [11]. Illumina HiSeq2500 150 bp paired end reads (BioProject PRJNA344486) were generated from *B. malayi* worms obtained from FR3 that were isolated 16 (L4), 30-, 42-, and 120-days post infection, corresponding to points in the progression from L4s through young adults to mature adults. Reads were mapped to the *B. malayi* reference genome using TopHat2 v2.1.1 [2] and counted on gene features using HTSeq v0.6.1p2 [3]. EdgeR v3.16.5 [4, 5] was used to identify differentially expressed genes in all pairwise comparisons with a *p* < 0.05 and an FDR < 0.05 cutoff for statistical significance. There were 2,753 male-biased genes and 3,109 female biased genes identified in the original differential expression analysis [10]. FPKMs and normalized FPKMs generated using Cufflinks and Cuffnorm, respectively, were subsequently used for visualization of differentially expressed genes.


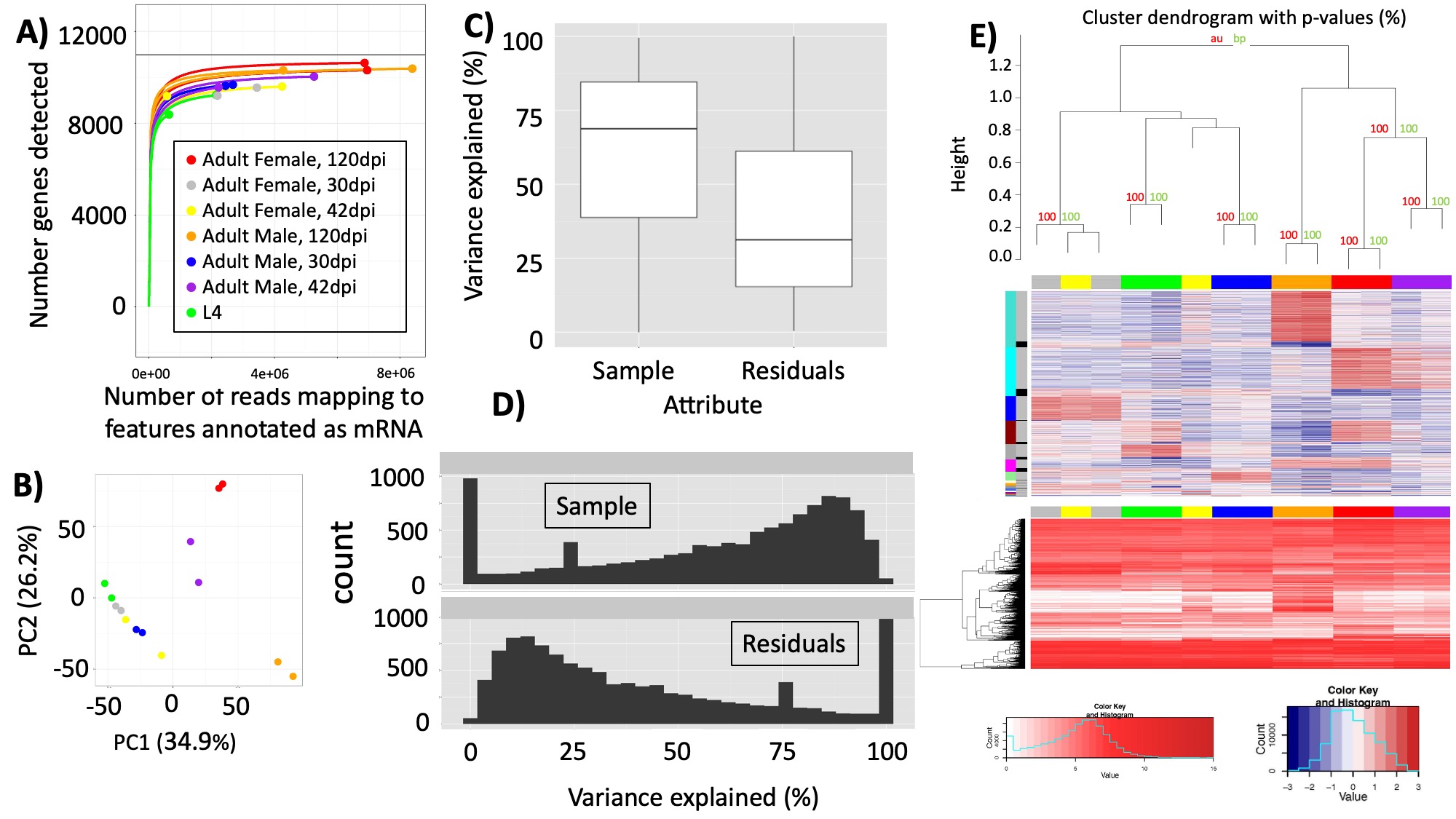


**Note: The heatmaps and PCA were generated using differentially expressed genes**

### Supplemental Figure 12: Differential expression reanalysis of *B. malayi* molting data reveals 6,038 differentially expressed genes. A) A rarefaction curve of the samples grouped by life stage B). Principal components analysis plot of the samples grouped by life stage. The PCA plot was generated using z-score normalized log_2_(TPM) values of the differentially expressed genes C) Linear mixed model assessing sample variation using varianceParition D) Histogram of the linear mixed model results E) Differential expression results using the z-score normalized log_2_(TPM) values of the differentially expressed genes in the top plot and the log_2_(TPM) values of the differentially expressed genes in the bottom plot. Both plots are ordered based on the Pvclust dendrogram, which was generated using the z-score normalized log_2_(TPM) values of the differentially expressed genes. The top annotation bar denotes the lifestage. In the top plot, the outer left annotation bar denotes the WGCNA cluster and the inner left annotation bar denotes whether the cluster matches the main expression profile (grey) or inverse expression profile (black).

## MACLEAN ET AL 2019 [12]: EFFECTS OF DIETHYLCARBAMAZINE AND IVERMECTIN TREATMENT ON *BRUGIA MALAYI* GENE EXPRESSION IN INFECTED GERBILS (*MERIONES UNGUICULATUS*)

Another drug study, looking at the effect of albendazole (Alb), diethylcarbamazine (DEC), ivermectin (IVM), and DMSO on the *B. malayi* transcriptome, identified differentially expressed genes based on drug treatment [12]. Adult male gerbils were injected intraperitoneally with 300 *B. malayi* L3 larvae and the worms were harvested 4 months post infection [12]. After treatment, half the gerbils were sacrificed 24 hours post treatment and the other half were sacrificed 7 days post treatment [12]. After RNA isolation and sequencing, the 150pb paired-end reads were mapped to the *B. malayi* WS253 reference genome using Tophat2 v2.0.14 [2] and counts were generated using HTSeq [3] based on gene features . Differentially expressed genes, using DESeq2 [13], were identified by normalizing count data, estimating dispersions, and then fitting a negative binomial model to the data [12]. Between 0 and 73 differentially expressed genes were originally identified in 18 pairwise comparisons. The *p-*values were adjusted to FDR values using the Benjamani-Hochberg method and the significance threshold was set at FDR < 0.05. Out of the total 203 differentially expressed genes identified, the majority were treatment-specific, with little overlap of differentially expressed genes across treatment


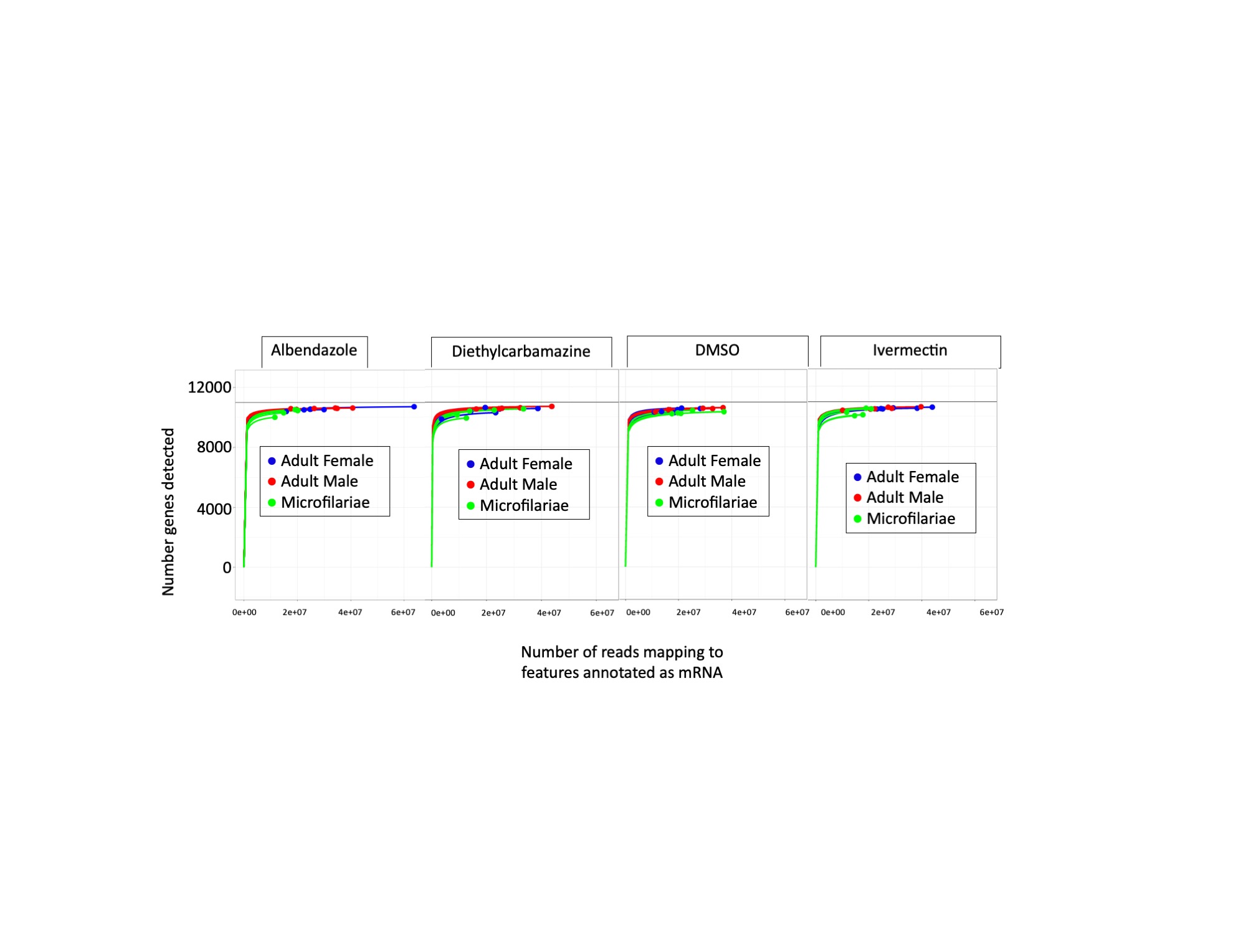


### Supplemental Figure 13: Rarefaction curves grouped by drug classification. Rarefaction curves of each sample grouped by drug classification with each sample colored by life stage


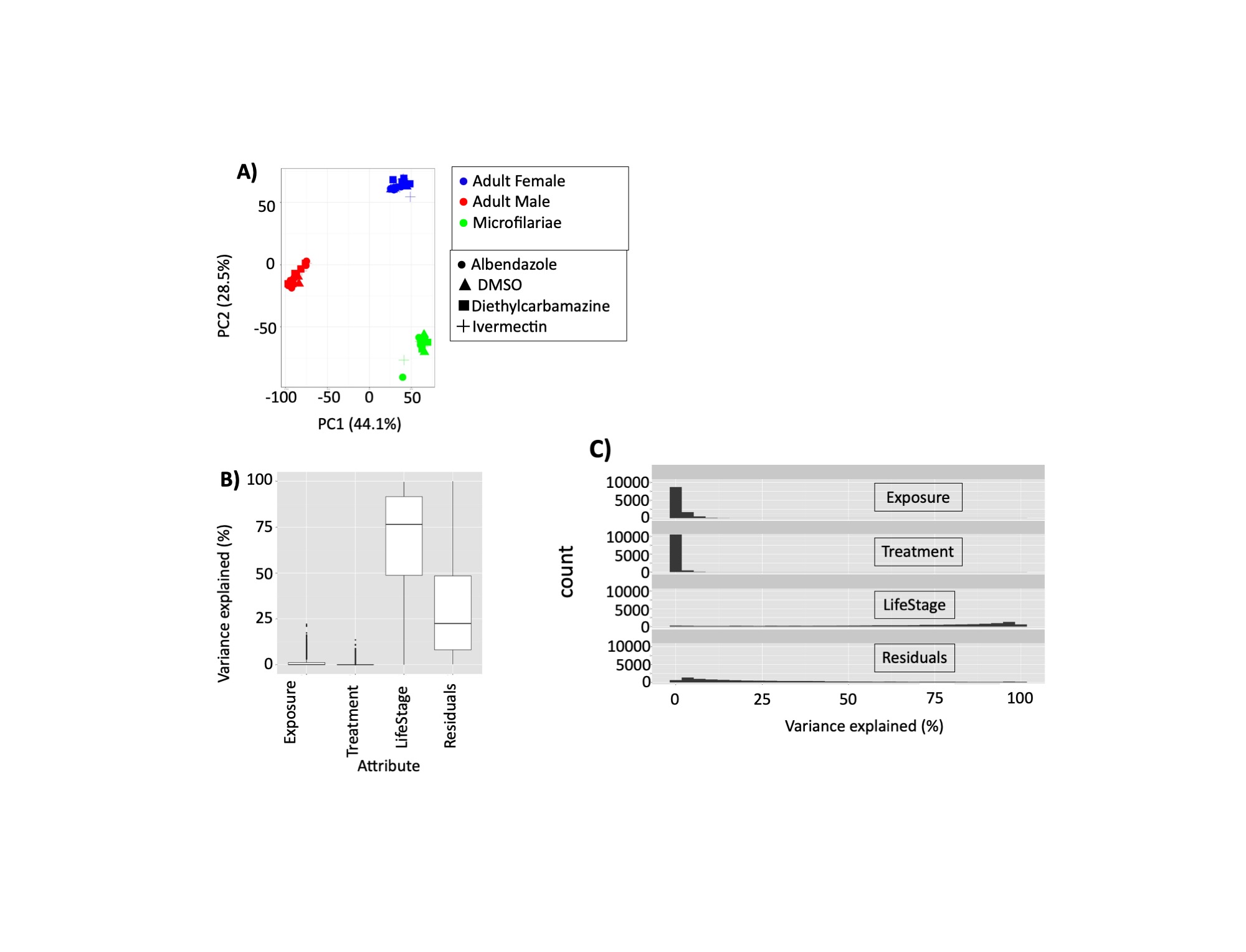


**Note: The PCA was generated using differentially expressed genes**

### Supplemental Figure 14: Variation in the Maclean drug study A) Principal components analysis plot of the samples grouped by life stage and drug treatment. The PCA plot was generated using z-score normalized log_2_(TPM) values of the differentially expressed genes B) Linear mixed model assessing sample variation using varianceParition C) Histogram of the linear mixed model results


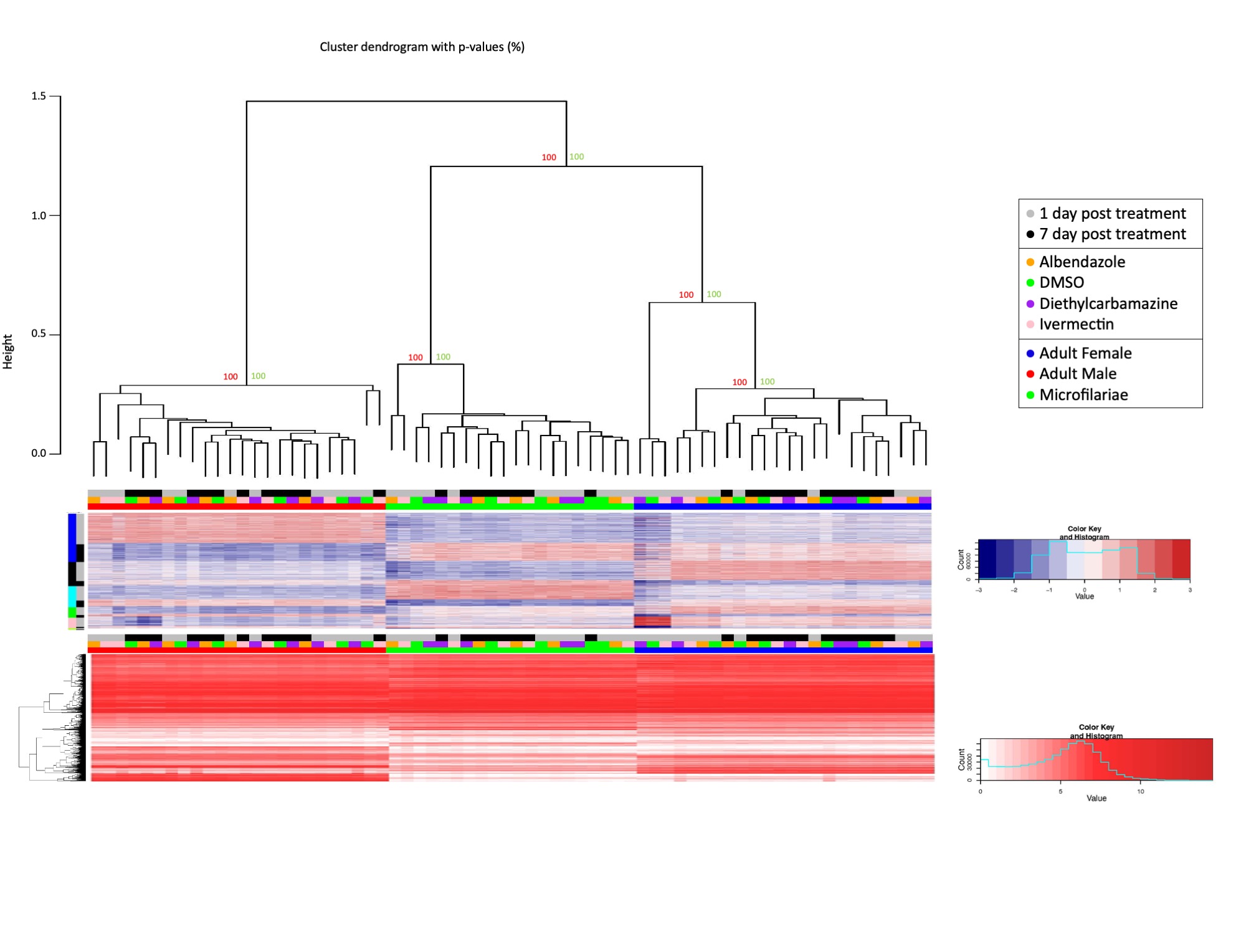


**Note: The heatmaps were generated using differentially expressed genes**

### Supplemental Figure 15: Differential expression reanalysis of *in vivo* drug treatment data reveals 9,350 differentially expressed genes. Differential expression results using the z-score normalized log_2_(TPM) values of the differentially expressed genes in the top plot and the log_2_(TPM) values of the differentially expressed genes in the bottom plot. Both plots are ordered based on the Pvclust dendrogram, which was generated using the z-score normalized log_2_(TPM) values of the differentially expressed genes. The top annotation bar denotes, from top to bottom, the days post treatment, drug treatment, and lifestage. In the top plot, the outer left annotation bar denotes the WGCNA cluster and the inner left annotation bar denotes whether the cluster matches the main expression profile (grey) or inverse expression profile (black).


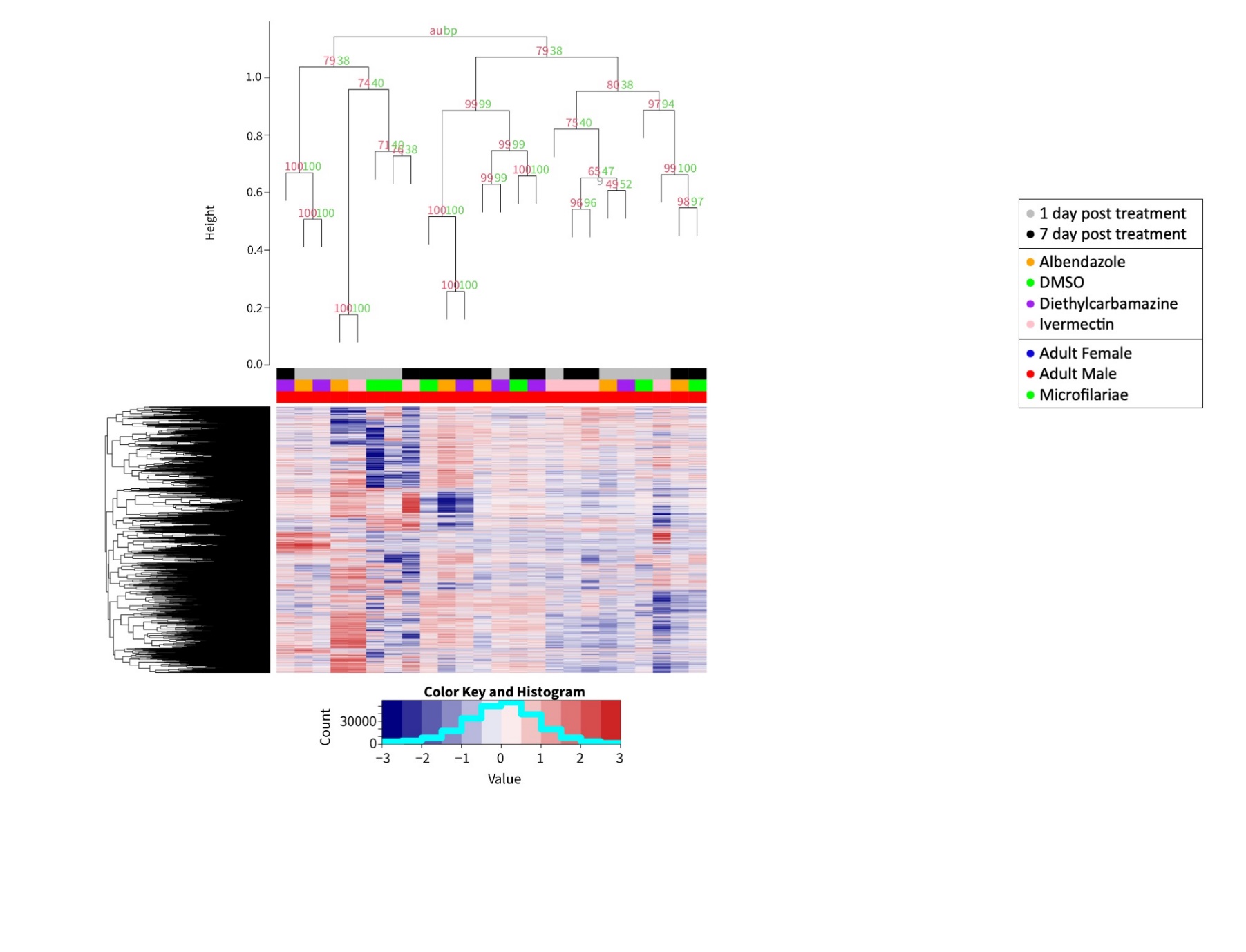

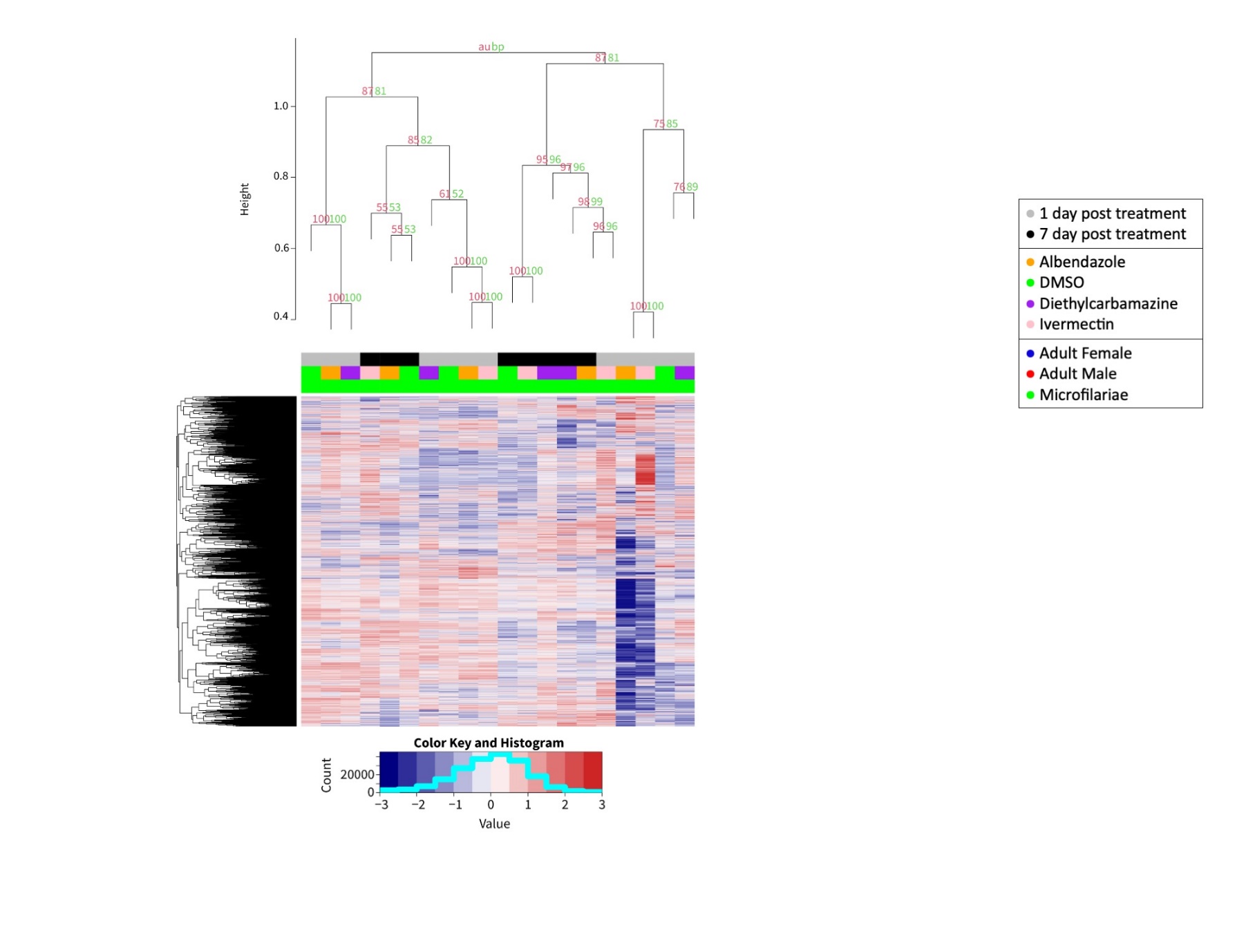

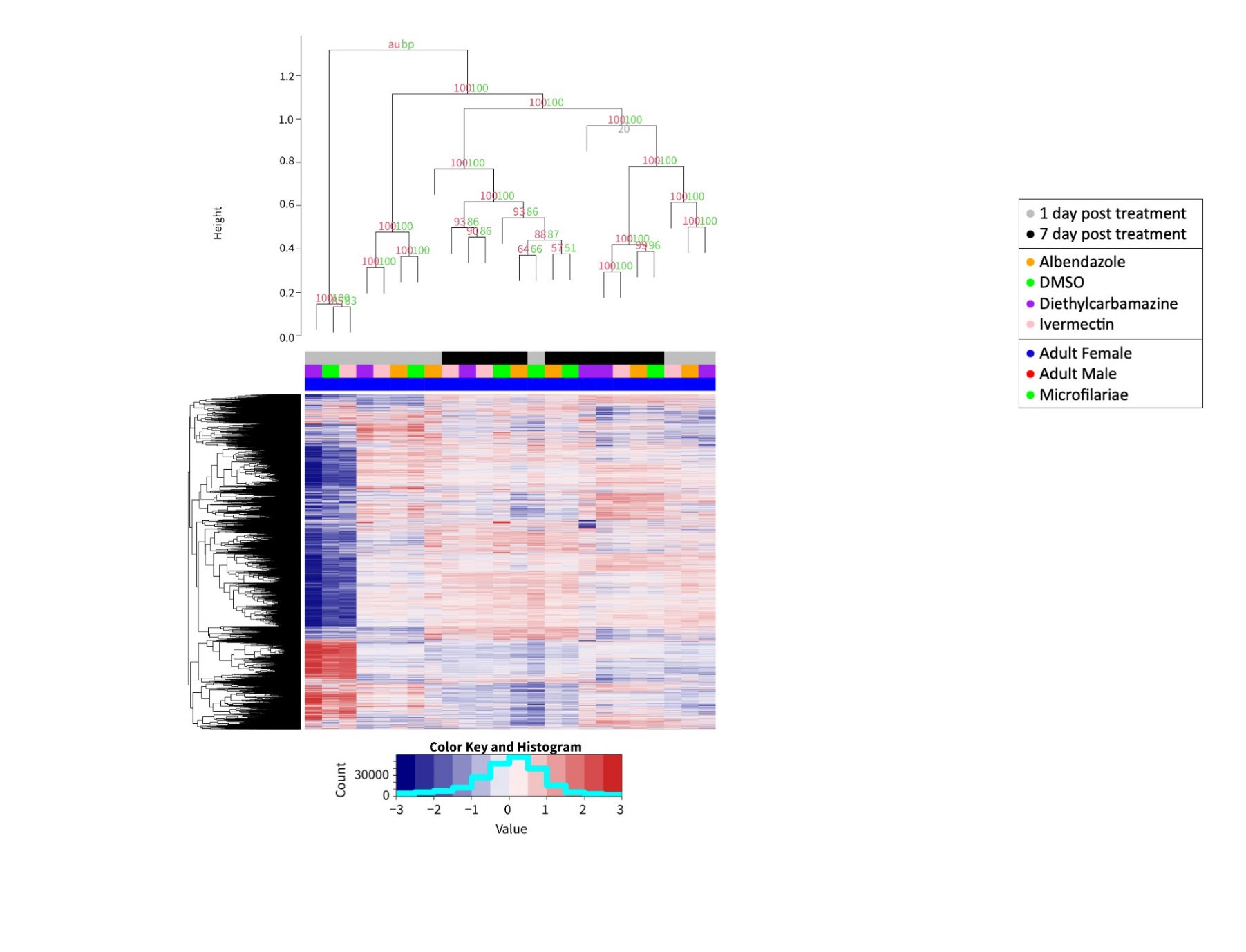


**C)**

**B)**

**A)**


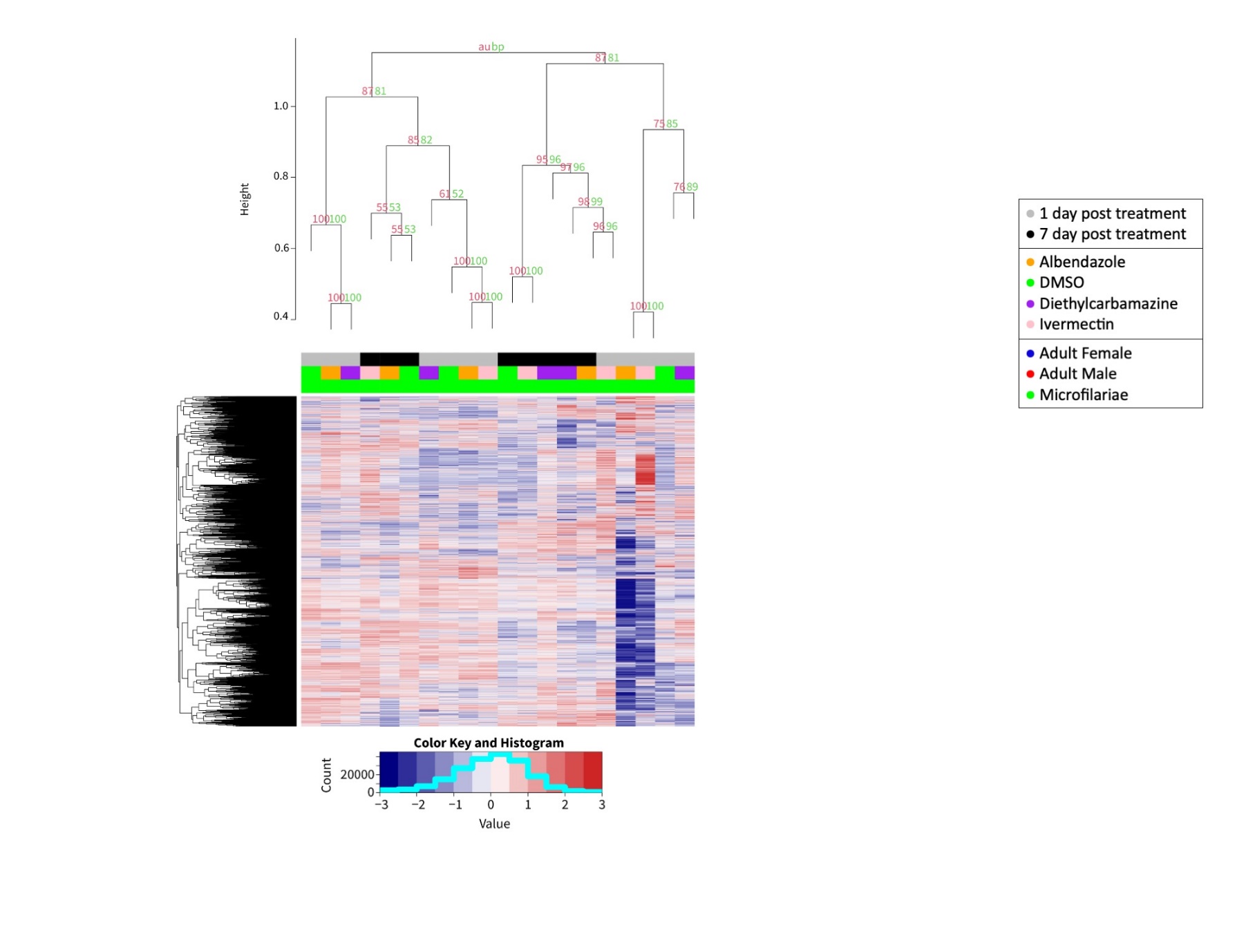


**Note: The heatmaps uses genes that passes the edgeR CPM filter**

### Supplemental Figure 16: Lack of Differentially Expressed Genes When Separated by Life Stage. A heatmap using the z-score normalized log_2_(TPM) values of the genes passing the CPM filter in the top plot and the log_2_(TPM) values of the genes passing the CPM filter in the bottom plot. Both plots are ordered based on the Pvclust dendrogram, which was generated using the z-score normalized log_2_(TPM) values of the genes passing the CPM filter. The top annotation bar denotes, from top to bottom, the days post treatment, drug treatment, and lifestage. The life stages are A) males, B) females, and C) microfilariae

## CHUNG ET AL 2019 [14, 15]: DRUG REPURPOSING OF BROMODOMAIN INHIBITORS AS POTENTIAL NOVEL THERAPEUTIC LEADS FOR LYMPHATIC FILARIASIS GUIDED BY MULTISPECIES TRANSCRIPTOMICS

In an in-depth study of the transcriptome of the *B. malayi* life cycle, discrete expression clusters were identified between the ten life cycle stages sequenced [14, 15]. Although this study considered both the *B. malayi* and the *w*Bm transcriptome, only the *B. malayi* transcriptome will be considered here as the *w*Bm transcriptome data has already been reanalyzed [11]. The RNA was isolated from samples belonging to the ten life stages, libraries created using the NEBNext Ultra Directional RNA Library Prep kit and polyA enrichment was performed using the NEBNext poly(A) mRNA magnetic isolation kit. The samples were then sequenced on an Illumina HiSeq2500, generating 100 bp paired end reads that were reversely stranded. The reads were aligned to the *B. malayi* WS259 genome using TopHat v1.4.0 [2] and counts were calculated using HTSeq v0.5.3p9 [3] using union mode on exon features. Five genes that were identified as overlapping with rRNAs were removed as well as the vector 18-hpi sample (due to poor number of reads). Using edgeR v3.20.1 [4, 5], genes were filtered based on a cpm requirement of at least 5 reads per gene in at least two samples. Genes that did not meet this requirement were removed. Genes were then fitted to a generalized linear log model and differential expression was assessed using a quasilikelihood F test. Significance was set a *p*-value < 0.05 and FDR < 0.05. There were 10,297 differentially expressed genes identified among the 16 life stages.


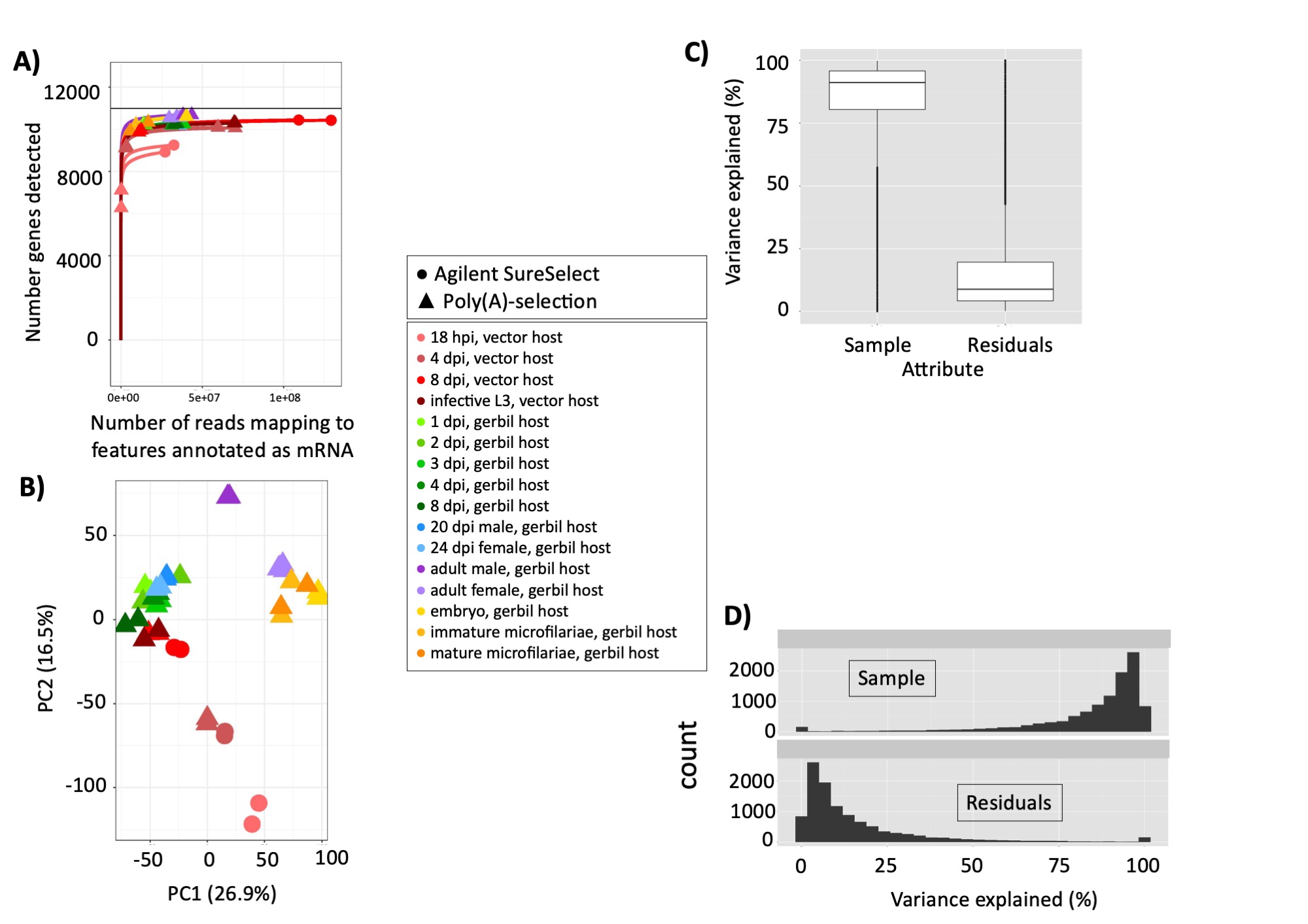


**Note: The PCA was generated using differentially expressed genes**

### Supplemental Figure 17: Reanalysis of Chung lifecycle data A) Rarefaction curve of the samples grouped by life stage B). Principal components analysis plot of the samples grouped by life stage. The PCA plot was generated using z-score normalized log_2_(TPM) values of the differentially expressed genes C) Linear mixed model assessing sample variation using varianceParition D) Histogram of the linear mixed model results


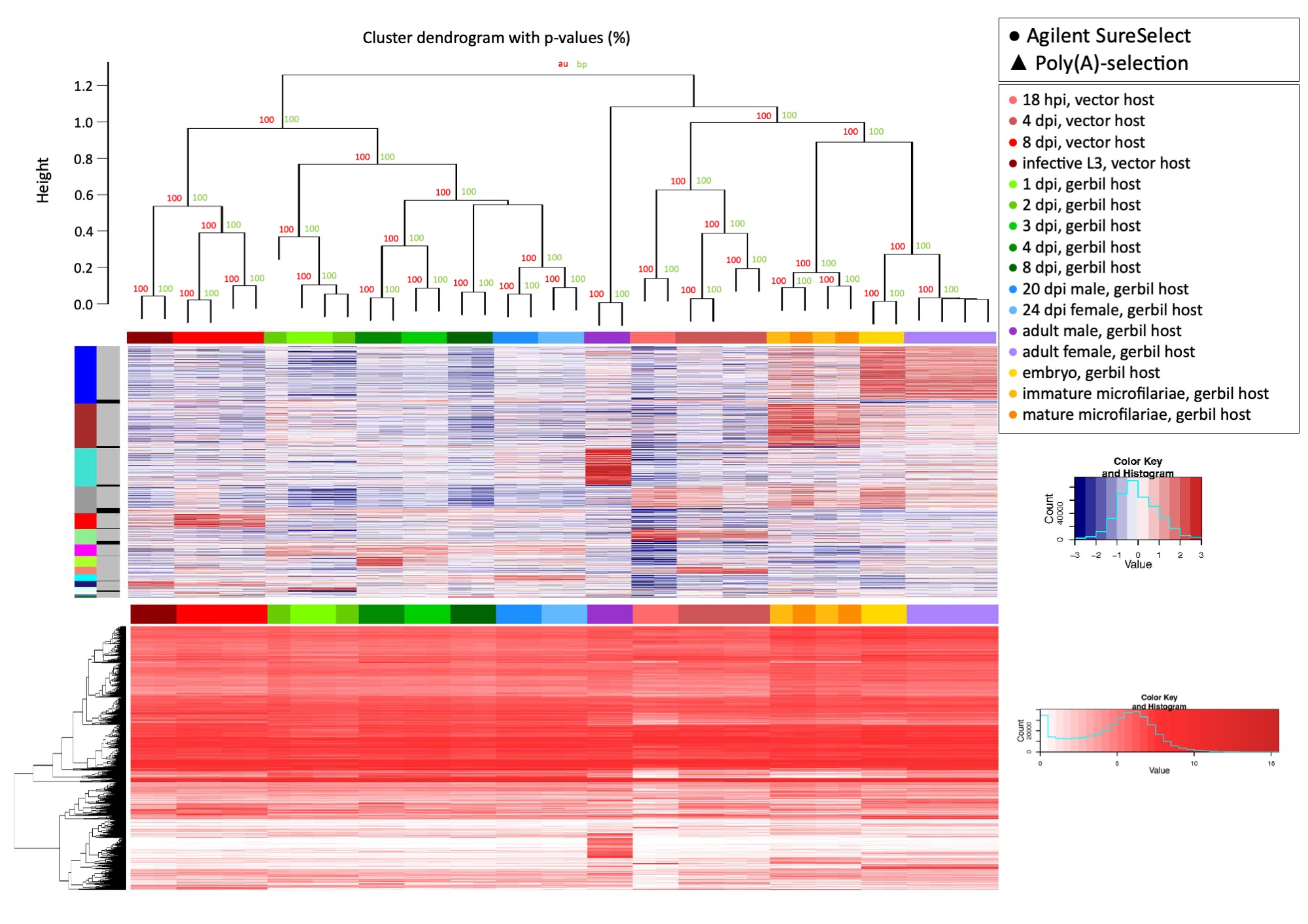


**Note: The heatmaps were generated using differentially expressed genes**

### Supplemental Figure 18: Differential expression reanalysis of *B. malayi* lifecycle data reveals 10,182 differentially expressed genes. Differential expression results using the z-score normalized log_2_(TPM) values of the differentially expressed genes in the top plot and the log_2_(TPM) values of the differentially expressed genes in the bottom plot. Both plots are ordered based on the Pvclust dendrogram, which was generated using the z-score normalized log_2_(TPM) values of the differentially expressed genes. The top annotation bar denotes the lifestage. In the top plot, the outer left annotation bar denotes the WGCNA cluster and the inner left annotation bar denotes whether the cluster matches the main expression profile (grey) or inverse expression profile (black).

## GROTE ET AL 2020 [16]: PREDICTION PIPELINE FOR DISCOVERY OF REGULATORY MOTIFS ASSOCIATED WITH *BRUGIA MALAYI* MOLTING

In order to assess *B. malayi* transcription factors and the role they play in regulatory development; a transcriptomics study was performed using L3 and L4 stage *B. malayi* [16]. This molt is the first molt occurring within the definitive host and plays and important role in the filarial infection life cycle. The specific life stages that were sequenced were the infective L3 larvae (isolated from *Aedes aegypti* mosquitos) and L3 larvae recovered from gerbils 6 days and 9 days post infection. The transcriptomic data for the L4 (14 dpi) larvae had been previously published [10]. The RNA was isolated and non-strand specific libraries were generated using the NEBNext Ultra II Library prep kit. The libraries were sequenced using the Illumina NextSeq500 which generated 150bp paired end reads. The resulting reads were mapped using the Tophat2 [2], and counts were generated using HTSeq [3]. Both DESeq [13] and EdgeR [4, 5] for the differential expression analysis, keeping overlapping genes with an FDR < 0.01. Stage-specific expression between the infective L3 larvae, 6-dpi and 9-dpi L3 larvae, and the L4 stage were identified.


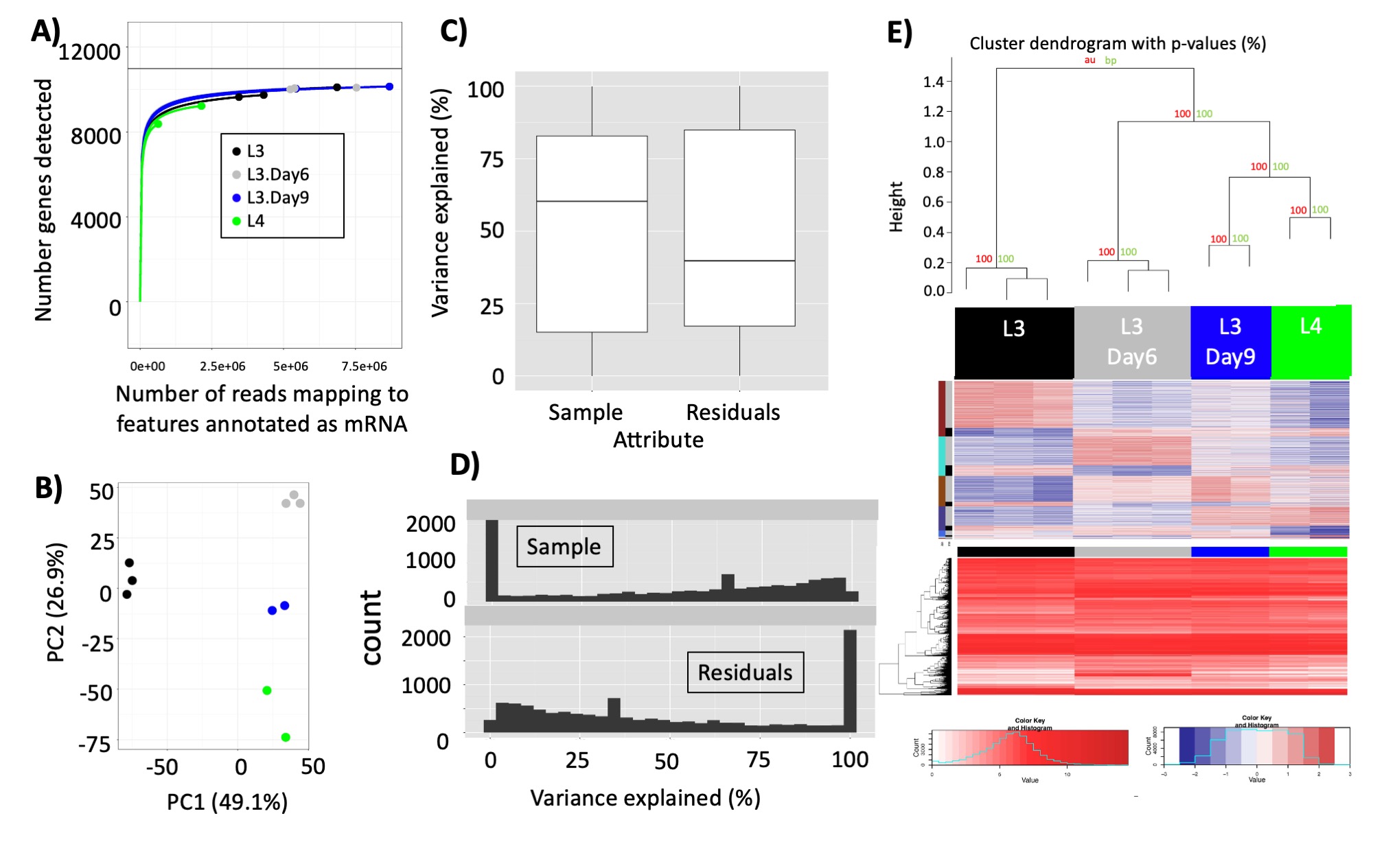


**Note: The heatmaps and PCA were generated using differentially expressed genes**

### Supplemental Figure 19: Differential expression reanalysis of *B. malayi* molting data reveals 5,227 differentially expressed genes. A) A rarefaction curve of the samples grouped by molt stage B). Principal components analysis plot of the samples grouped by molt stage. The PCA plot was generated using z-score normalized log_2_(TPM) values of the differentially expressed genes C) Linear mixed model assessing sample variation using varianceParition D) Histogram of the linear mixed model results E) Differential expression results using the z-score normalized log_2_(TPM) values of the differentially expressed genes in the top plot and the log_2_(TPM) values of the differentially expressed genes in the bottom plot. Both plots are ordered based on the Pvclust dendrogram, which was generated using the z-score normalized log_2_(TPM) values of the differentially expressed genes. The top annotation bar denotes the lifestage. In the top plot, the outer left annotation bar denotes the WGCNA cluster and the inner left annotation bar denotes whether the cluster matches the main expression profile (grey) or inverse expression profile (black).

## CHEVIGNON ET AL 2021 [17]: DUAL RNA-SEQ ANALYSES AT SOMA AND GERMLINE LEVELS REVEAL EVOLUTIONARY INNOVATIONS IN THE ELEPHANTIASIS-AGENT *BRUGIA MALAYI*, AND ADAPTATION OF ITS *WOLBACHIA* ENDOSYMBIONT

A transcriptomics study of the adult female *B. malayi* germline and soma tissues was performed to further understand the mutualism between the nematode and its obligate *Wolbachia* endosymbiont (*w*Bm) [17]. Samples from three sites were picked: the proliferative zone (PZ), the meiotic zone (MZ), and the body wall (BW) [17]. The samples from these three sites (three biological replicates per site) were sequenced on an Illumina machine with 75pb paired-end reads generated. The quality of these reads was assessed with FASTQC v0.11.7 [18] were then aligned to the WS265 *B. malayi* reference genome using HiSAT2 v2.1.0 [19]. Counts were generated using featureCounts from the subread v1.6.2 package (counting on CDS or rRNA feature) [17]. Between 1,285 and 2,739 differentially expressed genes were identified using the DESeq2 v1.22.2 pipeline [13] with a threshold set of FDR < 0.01 and log_2_ fold change of +/- 2 in these three pairwise comparisons.


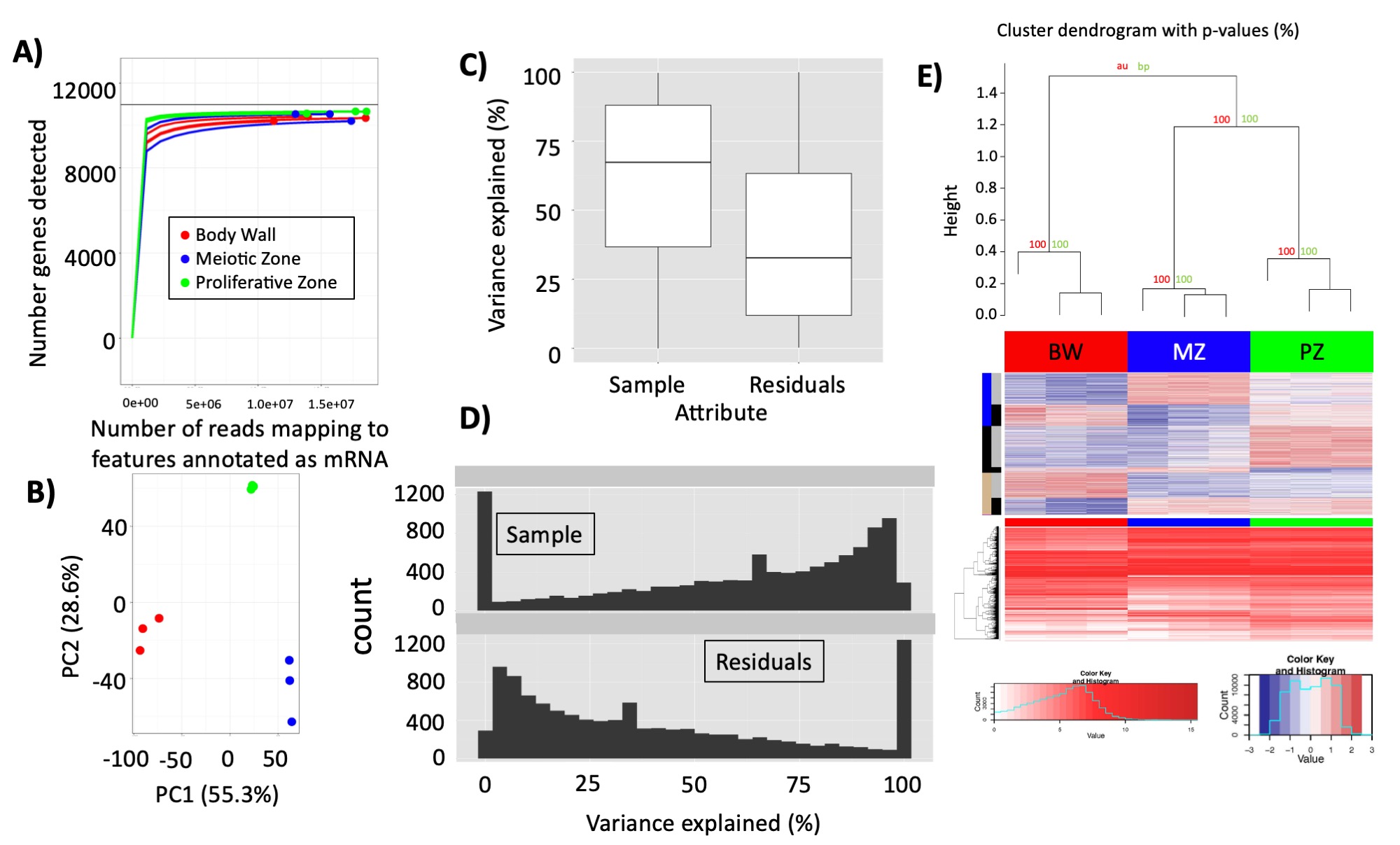


**Note: The heatmaps and PCA were generated using differentially expressed genes**

### Supplemental Figure 20: Differential expression reanalysis of soma and germline data reveals 7,191 differentially expressed genes. A) A rarefaction curve of the samples grouped by sample sites B). Principal components analysis plot of the samples grouped by sample site. The PCA plot was generated using z-score normalized log_2_(TPM) values of the differentially expressed genes C) Linear mixed model assessing sample variation using varianceParition D) Histogram of the linear mixed model results E) Differential expression results using the z-score normalized log_2_(TPM) values of the differentially expressed genes in the top plot and the log_2_(TPM) values of the differentially expressed genes in the bottom plot. Both plots are ordered based on the Pvclust dendrogram, which was generated using the z-score normalized log_2_(TPM) values of the differentially expressed genes. The top annotation bar denotes the sample site. In the top plot, the outer left annotation bar denotes the WGCNA cluster and the inner left annotation bar denotes whether the cluster matches the main expression profile (grey) or inverse expression profile (black).

## QUEK ET AL 2022 [20]: *WOLBACHIA* DEPLETION BLOCKS TRANSMISSION OF LYMPHATIC FILARIASIS BY PREVENTING CHITINASE-DEPENDENT PARASITE EXSHEATHMENT

An important target in the eradication of certain filarial infections is the obligate *Wolbachia* endosymbiont. A study investigated the effect of treating *B. malayi* microfilariae with a tetracycline to understand how the *Wolbachia*-depletion affects the microfilariae development [20]. Microfilariae were isolated from intraperitoneally infected male gerbils who had been treated with tetracycline hydrochloride, rifampicin, or AWZ1066. The samples that were used for RNA-Sequencing were ones from a control dataset or where the host gerbils had undergone a 6-week tetracycline treatment. RNA was isolated from the microfilariae and sequenced on an Illumina HiSeq 4000, generating 150bp paired end reads. These filtered reads were aligned to the *B. malayi* reference genome using the Subread-Aligner’s Subjunc program. Counts were generated using FeatureCounts and differential expression assessed using edgeR. Low expressed genes were filtered using edgeR’s *filterByExpr* function and quasi-likelihood F (QLF) test was used to identify differentially expressed genes. There were 1,079 differentially expressed genes identified between the analysis and 6-week treated microfilariae.


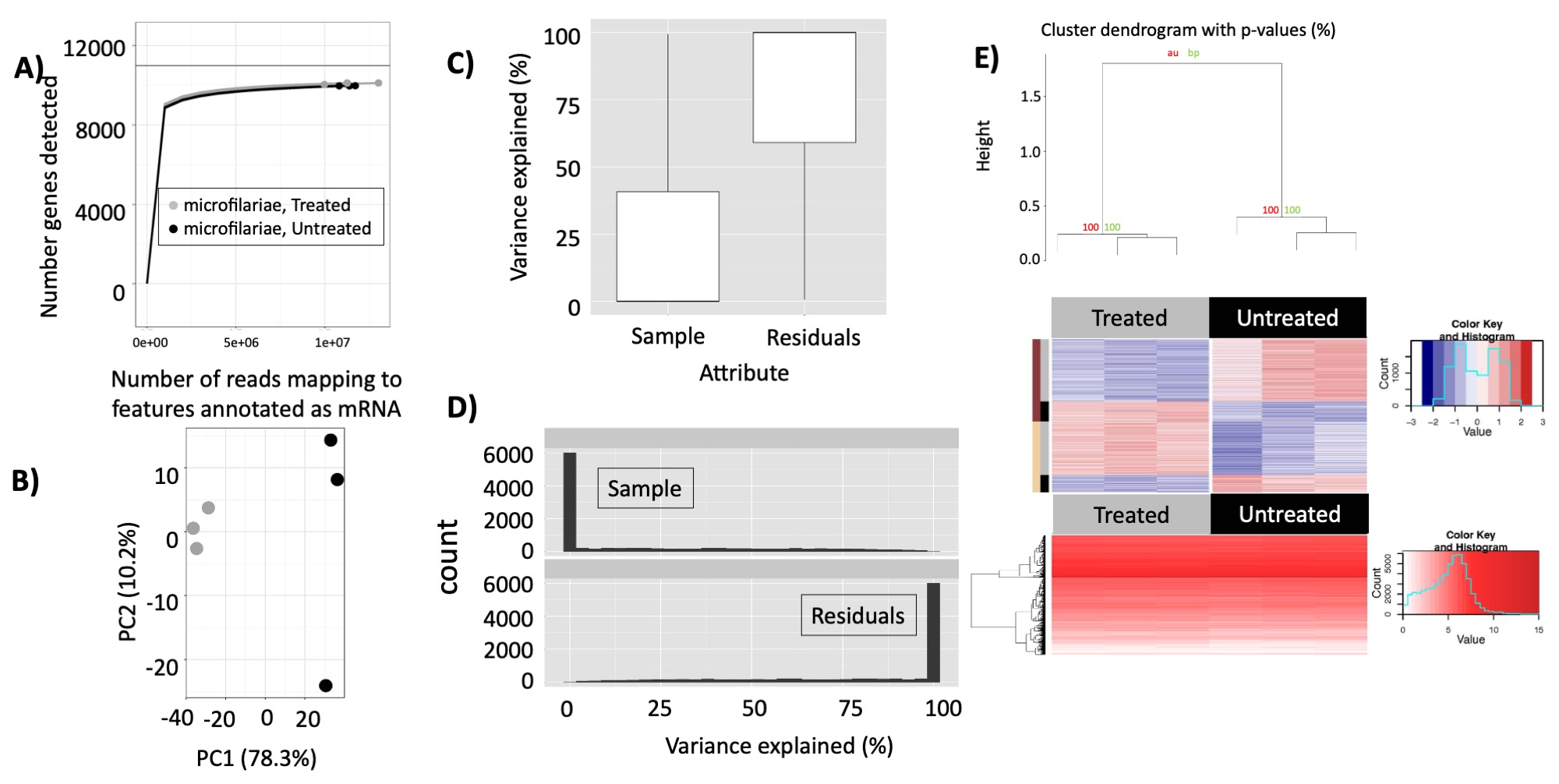


**Note: The heatmaps and PCA were generated using differentially expressed genes**

### Supplemental Figure 21: Differential expression reanalysis of tetracycline-treated *B. malayi* reveals 1,423 differentially expressed genes. A) A rarefaction curve of the samples grouped by treatment B). Principal components analysis plot of the samples grouped by treatment. The PCA plot was generated using z-score normalized log_2_(TPM) values of the differentially expressed genes C) Linear mixed model assessing sample variation using varianceParition D) Histogram of the linear mixed model results E) Differential expression analysis reveals 1.423 results using the z-score normalized log_2_(TPM) values of the differentially expressed genes in the top plot and the log_2_(TPM) values of the differentially expressed genes in the bottom plot. Both plots are ordered based on the Pvclust dendrogram, which was generated using the z-score normalized log_2_(TPM) values of the differentially expressed genes. The top annotation bar denotes the treatment group. In the top plot, the outer left annotation bar denotes the WGCNA cluster and the inner left annotation bar denotes whether the cluster matches the main expression profile (grey) or inverse expression profile (black).

## AIRS ET AL 2022 [21]: SPATIAL TRANSCRIPTOMICS REVEALS ANTIPARASITIC TARGETS ASSOCIATED WITH ESSENTIAL BEHAVIOURS IN THE HUMAN PARASITE *BRUGIA MALAYI*

In 2022, a spatial transcriptomics study was performed in order to assess the gene expression across important tissues within *B. mala*yi with the aim of identifying antiparasitic drug targets [21]. Adult female *B. malayi* nematodes were dissected, separating the head region from the body region. The purpose of this design is to identify gene targets enriched in the head tissues of the nematode. RNA was isolated and sequenced on an Illumina HiSeq2500, generating single end 1x100bp reads. The RNA-Seq reads were mapped to the *B. malayi* reference genome, and gene counts generated, using STAR [22]. DESeq2 [13] was used to identify differentially expressed genes. In the original analysis, there were 2,406 differentially expressed genes enriched in the head tissue, as well as that important filarial antigens are enriched in head tissue.


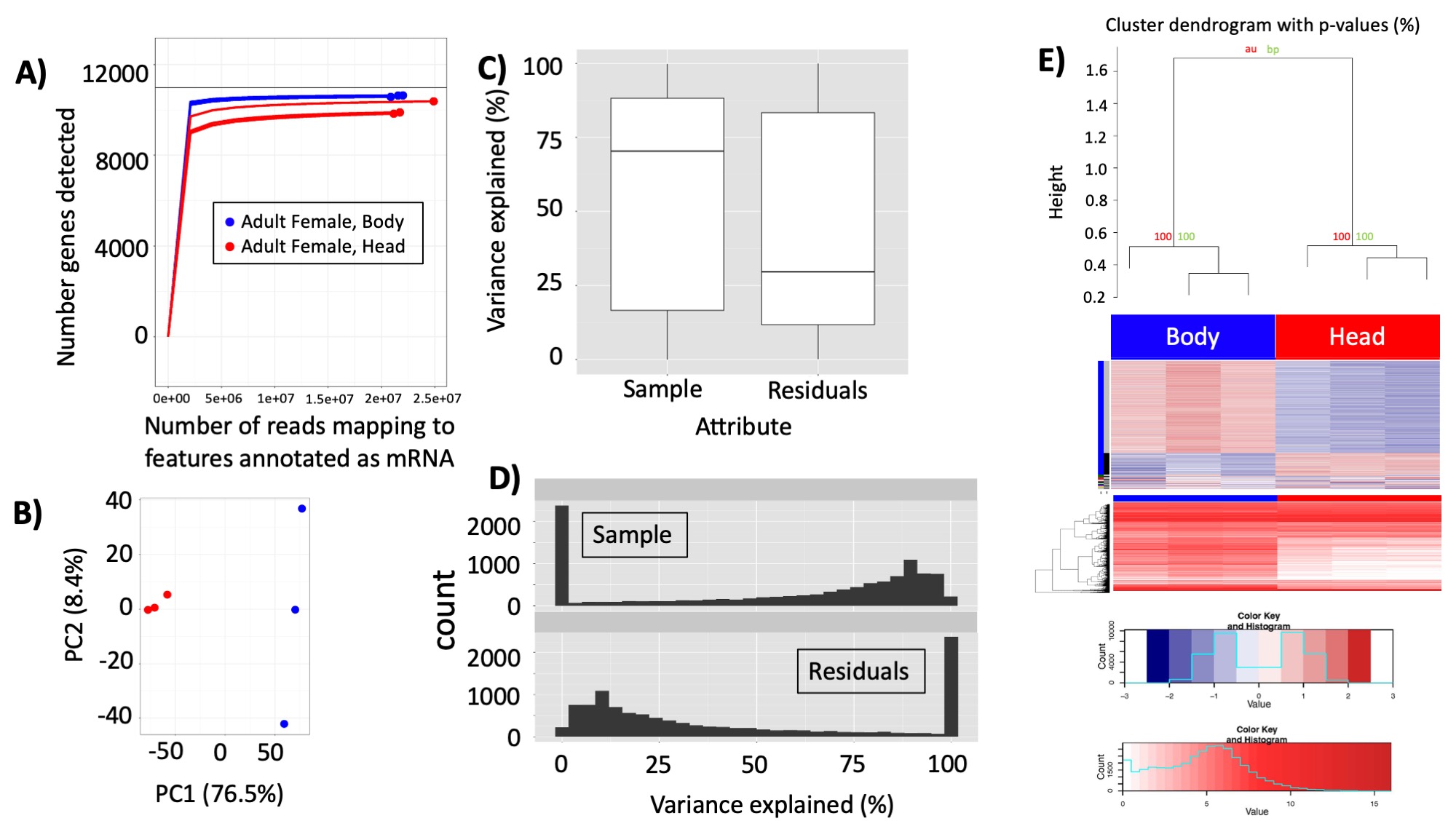


**Note: The heatmaps and PCA were generated using differentially expressed genes**

### Supplemental Figure 22: Differential expression reanalysis of *B. malayi* body site data reveals 6,256 differentially expressed genes. A) A rarefaction curve of the samples grouped by body site B). Principal components analysis plot of the samples grouped by body site. The PCA plot was generated using z-score normalized log_2_(TPM) values of the differentially expressed genes C) Linear mixed model assessing sample variation using varianceParition D) Histogram of the linear mixed model results E) Differential expression results using the z-score normalized log_2_(TPM) values of the differentially expressed genes in the top plot and the log_2_(TPM) values of the differentially expressed genes in the bottom plot. Both plots are ordered based on the Pvclust dendrogram, which was generated using the z-score normalized log_2_(TPM) values of the differentially expressed genes. The top annotation bar denotes the body site. In the top plot, the outer left annotation bar denotes the WGCNA cluster and the inner left annotation bar denotes whether the cluster matches the main expression profile (grey) or inverse expression profile (black).

## REFERENCES

1. Choi, Y.J., et al., *A deep sequencing approach to comparatively analyze the transcriptome of lifecycle stages of the filarial worm, Brugia malayi.* PLoS Negl Trop Dis, 2011. **5**(12): p. e1409.

2. Kim, D., et al., *TopHat2: accurate alignment of transcriptomes in the presence of insertions, deletions and gene fusions.* Genome Biol, 2013. **14**(4): p. R36.

3. Anders, S., P.T. Pyl, and W. Huber, *HTSeq--a Python framework to work with high-throughput sequencing data.* Bioinformatics, 2015. **31**(2): p. 166-9.

4. Chen, Y., et al., *edgeR: differential analysis of sequence read count data.* 2023.

5. Robinson, M.D., D.J. McCarthy, and G.K. Smyth, *edgeR: a Bioconductor package for differential expression analysis of digital gene expression data.* Bioinformatics, 2010. **26**(1): p. 139-40.

6. Ballesteros, C., et al., *The Effects of Ivermectin on Brugia malayi Females In Vitro: A Transcriptomic Approach.* PLoS Negl Trop Dis, 2016. **10**(8): p. e0004929.

7. BroadInstitute. *Picard*. [cited 2022; Available from: <http://broadinstitute.github.io/picard/>.

8. Ballesteros, C., et al., *The Effect of In Vitro Cultivation on the Transcriptome of Adult Brugia malayi.* PLoS Negl Trop Dis, 2016. **10**(1): p. e0004311.

9. Libro, S., B.E. Slatko, and J.M. Foster, *Characterization of innate immunity genes in the parasitic nematode Brugia malayi.* Symbiosis, 2016. **68**: p. 145-155.

10. Grote, A., et al., *Defining Brugia malayi and Wolbachia symbiosis by stage-specific dual RNA-seq.* PLoS Negl Trop Dis, 2017. **11**(3): p. e0005357.

11. Chung, M., et al., *A Meta-Analysis of Wolbachia Transcriptomics Reveals a Stage-Specific Wolbachia Transcriptional Response Shared Across Different Hosts.* G3 (Bethesda), 2020. **10**(9): p. 3243-3260.

12. Maclean, M.J., et al., *Effects of diethylcarbamazine and ivermectin treatment on Brugia malayi gene expression in infected gerbils (Meriones unguiculatus).* Parasitol Open, 2019. **5**.

13. Love, M.I., W. Huber, and S. Anders, *Moderated estimation of fold change and dispersion for RNA-seq data with DESeq2.* Genome Biol, 2014. **15**(12): p. 550.

14. Chung, M., et al., *Drug Repurposing of Bromodomain Inhibitors as Potential Novel Therapeutic Leads for Lymphatic Filariasis Guided by Multispecies Transcriptomics.* mSystems, 2019. **4**(6).

15. Chung, M., et al., *Multispecies Transcriptomics Data Set of Brugia malayi, Its Wolbachia Endosymbiont wBm, and Aedes aegypti across the B. malayi Life Cycle.* Microbiol Resour Announc, 2018. **7**(18).

16. Grote, A., et al., *Prediction pipeline for discovery of regulatory motifs associated with Brugia malayi molting.* PLoS Negl Trop Dis, 2020. **14**(6): p. e0008275.

17. Chevignon, G., et al., *Dual RNAseq analyses at soma and germline levels reveal evolutionary innovations in the elephantiasis-agent Brugia malayi, and adaptation of its Wolbachia endosymbionts.* PLoS Negl Trop Dis, 2021. **15**(1): p. e0008935.

18. Andrews, S. *FASTQC: a quality control tool for high throughput sequence data*. Available from: <http://www.bioinformatics.babraham.ac.uk/projects/fastqc/>.

19. Kim, D., et al., *Graph-based genome alignment and genotyping with HISAT2 and HISAT-genotype.* Nat Biotechnol, 2019. **37**(8): p. 907-915.

20. Quek, S., et al., *Wolbachia depletion blocks transmission of lymphatic filariasis by preventing chitinase-dependent parasite exsheathment.* Proc Natl Acad Sci U S A, 2022. **119**(15): p. e2120003119.

21. Airs, P.M., et al., *Spatial transcriptomics reveals antiparasitic targets associated with essential behaviors in the human parasite Brugia malayi.* PLoS Pathog, 2022. **18**(4): p. e1010399.

22. Dobin, A., et al., *STAR: ultrafast universal RNA-seq aligner.* Bioinformatics, 2013. **29**(1): p. 15-21.
