# Supplementary figures and images for: Updated annotation and meta-analysis of Brugia malayi transcriptomics data reveals consistent transcriptional profiles across time and space with some study-specific differences in adult female worm transcriptional profiles
Source: PLoS Negl Trop Dis. 2024 Sep 26;18(9):e0012511. doi: 10.1371/journal.pntd.0012511 (PMC11460672; doi:10.1371/journal.pntd.0012511)

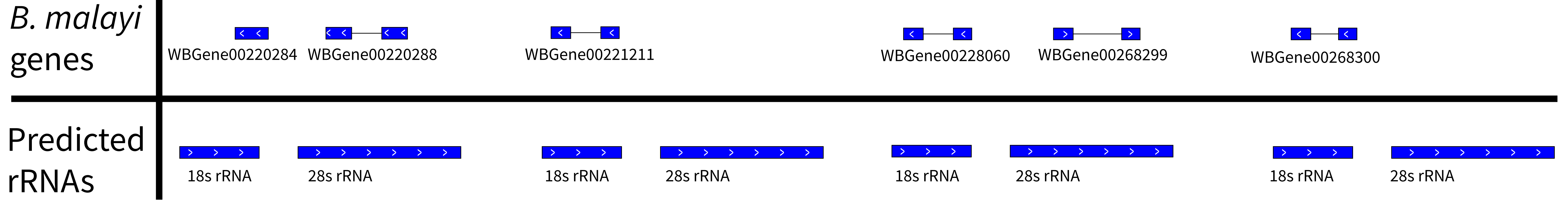

Supplement: S1 Fig — An adapted schematic of annotated B. malayi genes overlapping predicted rRNA features from IGV. The blue boxes denote exons with white arrows showing the respective strand. All gene locations and sizes are not to scale. (PDF) [file pntd.0012511.s005.pdf]

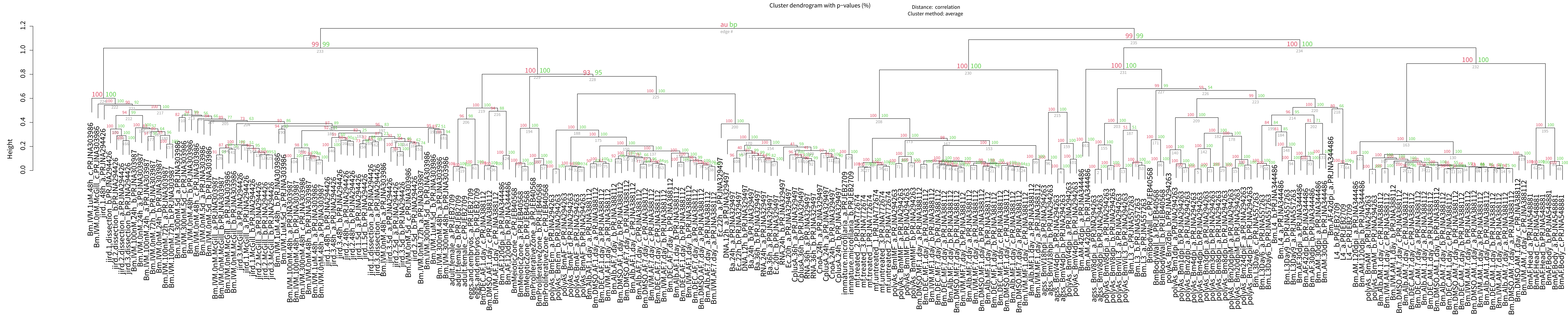

Supplement: S3 Fig — The pvclust dendrogram, provided in Fig 1, with all sample names and support values shown. The red values are approximately unbiased (au) and the green are the bootstrap support (bp) values. The size of support values above a height of 0.6 have been increased for illustrative purposes. The sample names are included in the dendrogram. (PDF) [file pntd.0012511.s007.pdf]
