# Supplementary material for: Updated annotation and meta-analysis of Brugia malayi transcriptomics data reveals consistent transcriptional profiles across time and space with some study-specific differences in adult female worm transcriptional profiles
Source: PLoS Negl Trop Dis. 2024 Sep 26;18(9):e0012511. doi: 10.1371/journal.pntd.0012511 (PMC11460672; doi:10.1371/journal.pntd.0012511)
Supplement: S2 Fig — An adapted version of Fig 1 with the sample names at the top of each column without the dendrogram. The sample names are from the dendrogram presented in S3 Fig. The histogram at the bottom shows the distribution of all the z-score values in the heatmap. The heatmap uses a z-score normalization of log2(TPM) values for 9,727 differentially expressed genes between 237 samples reanalyzed from 12 projects. The legend at the top is broken into three sections: project color, if the sample was drug treated, and sample life stage. The left hand legend is broken into two sections: the outer section denotes the WGCNA cluster and the inner section denotes if cluster matches the main or inverse WGCNA cluster expression pattern. Samples were labeled with first author, title, and bioproject from [18–29]. (PDF) [file pntd.0012511.s006.pdf]

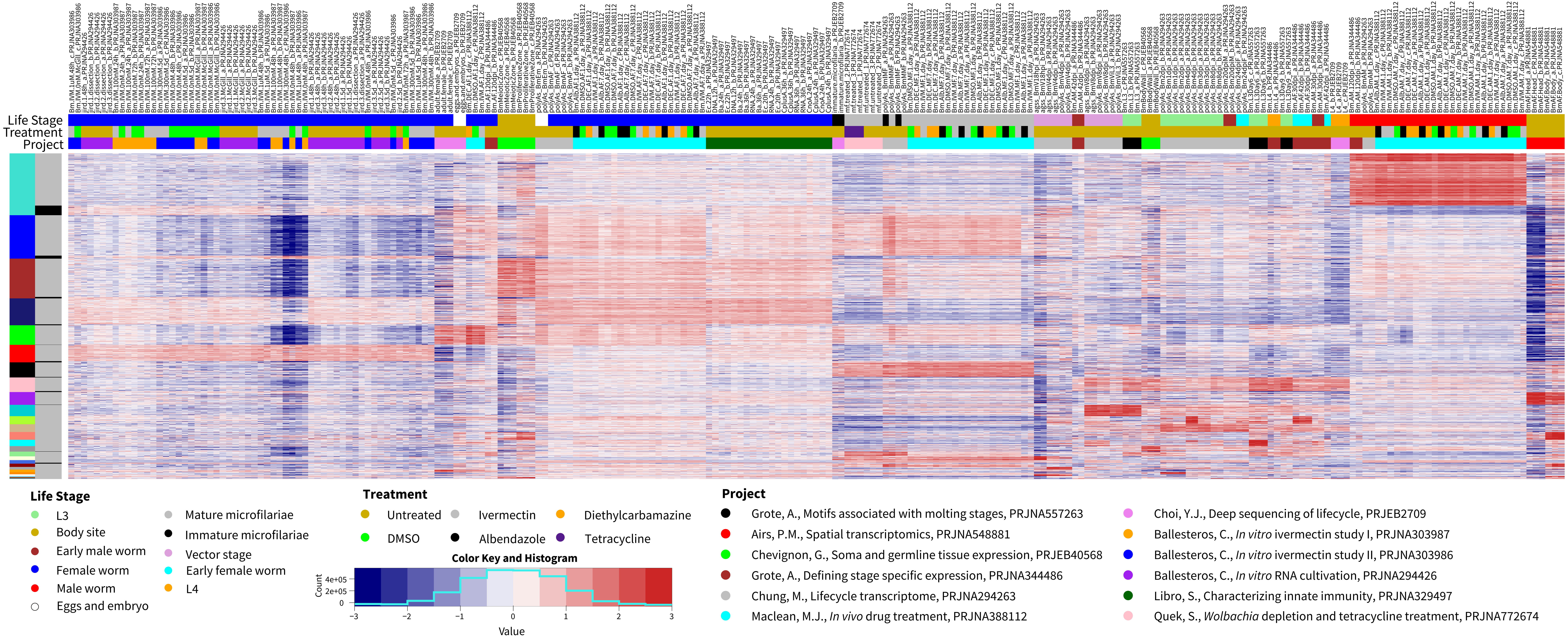

Life Stage
Treatment
Project
Life Stage
L3
Body site
Early male worm
Female worm
Male worm
Eggs and embryo
Mature microfilariae
Immature microfilariae
Vector stage
Early female worm
L4
Treatment
Untreated
Ivermectin
Diethylcarbamazine
DMSO
Albendazole
Tetracycline
Color Key and Histogram
Count
4e+05
2e+05
0
-3
-2
-1
0
1
2
3
Value
Project
Grote, A., Motifs associated with molting stages, PRJNA557263
Airs, P.M., Spatial transcriptomics, PRJNA548881
Chevignon, G., Soma and germline tissue expression, PRJEB40568
Grote, A., Defining stage specific expression, PRJNA344486
Chung, M., Lifecycle transcriptome, PRJNA294263
Maclean, M.J., *In vivo* drug treatment, PRJNA388112
Choi, Y.J., Deep sequencing of lifecycle, PRJEB2709
Ballesteros, C., *In vitro* ivermectin study I, PRJNA303987
Ballesteros, C., *In vitro* ivermectin study II, PRJNA303986
Ballesteros, C., *In vitro* RNA cultivation, PRJNA294426
Libro, S., Characterizing innate immunity, PRJNA329497
Quek, S., *Wolbachia* depletion and tetracycline treatment, PRJNA772674
