# Supplementary material for: Updated annotation and meta-analysis of Brugia malayi transcriptomics data reveals consistent transcriptional profiles across time and space with some study-specific differences in adult female worm transcriptional profiles
Source: PLoS Negl Trop Dis. 2024 Sep 26;18(9):e0012511. doi: 10.1371/journal.pntd.0012511 (PMC11460672; doi:10.1371/journal.pntd.0012511)
Supplement: S5 Fig — The dendrogram at the top of the heatmap was generated using pvclust. The red values are approximately unbiased (au) and the green are the bootstrap support (bp) values, both of which are generated by pvclust. The values shown are for illustrative purposes. The samples included are select adult female samples. The heatmap uses a z-score normalization of log2(TPM) values for the 7,785 differentially expressed genes. The legend at the top is broken into three sections: project color, if the sample was drug treated, and sample life stage. The left hand legend is broken into two sections: the outer section denotes the WGCNA cluster and the inner section denotes if cluster matches the main or inverse WGCNA cluster expression pattern. Samples labeled with first author, title, and bioproject from [19,20,23–25] (PDF) [file pntd.0012511.s009.pdf]

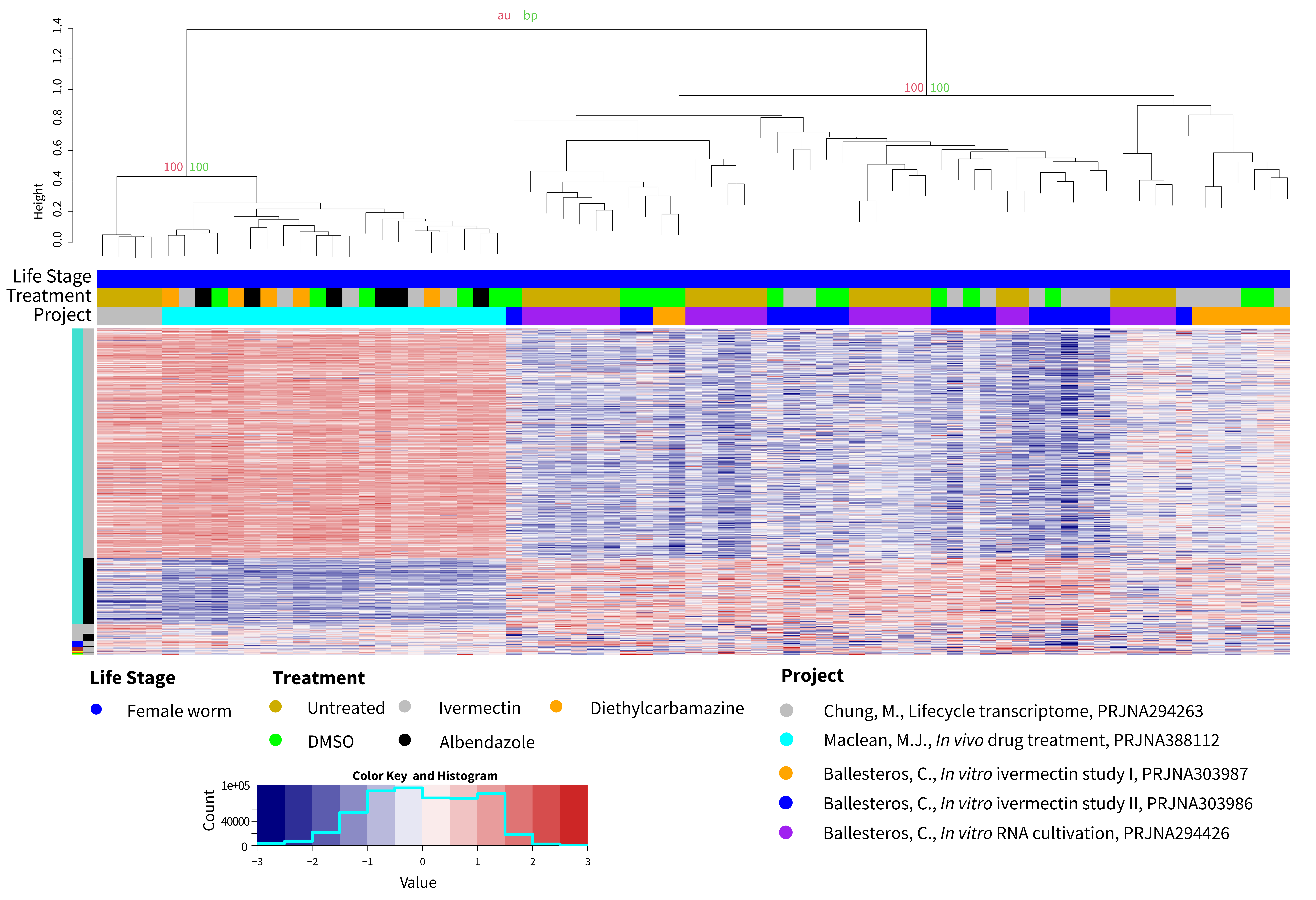
au bp
100 100
100 100
Height
0.0
0.2
0.4
0.6
0.8
1.0
1.2
1.4
Life Stage
Treatment
Project
Life Stage
Female worm
Treatment
Untreated
Ivermectin
Diethylcarbamazine
DMSO
Albendazole
Project
Chung, M., Lifecycle transcriptome, PRJNA294263
Maclean, M.J., *In vivo* drug treatment, PRJNA388112
Ballesteros, C., *In vitro* ivermectin study I, PRJNA303987
Ballesteros, C., *In vitro* ivermectin study II, PRJNA303986
Ballesteros, C., *In vitro* RNA cultivation, PRJNA294426
Color Key and Histogram
Count
1e+05
40000
0
−3
−2
−1
0
1
2
3
Value
