# Supplementary material for: Updated annotation and meta-analysis of Brugia malayi transcriptomics data reveals consistent transcriptional profiles across time and space with some study-specific differences in adult female worm transcriptional profiles
Source: PLoS Negl Trop Dis. 2024 Sep 26;18(9):e0012511. doi: 10.1371/journal.pntd.0012511 (PMC11460672; doi:10.1371/journal.pntd.0012511)
Supplement: S6 Fig — The dendrogram at the top of the heatmap was generated using pvclust. The red values are approximately unbiased (au) and the green are the bootstrap support (bp) values, both of which are generated by pvclust. The values shown are for illustrative purposes. The samples included are select adult female samples. The heatmap uses a z-score normalization of log2(TPM) values for the 2,906 differentially expressed genes. The legend at the top is broken into four sections: project color, if the sample was drug treated, sample life stage, and media status. The left hand legend is broken into two sections: the outer section denotes the WGCNA cluster and the inner section denotes if cluster matches the main or inverse WGCNA cluster expression pattern. Samples were labeled with first author, title, and bioproject from [18–20,22–25]. (PDF) [file pntd.0012511.s010.pdf]

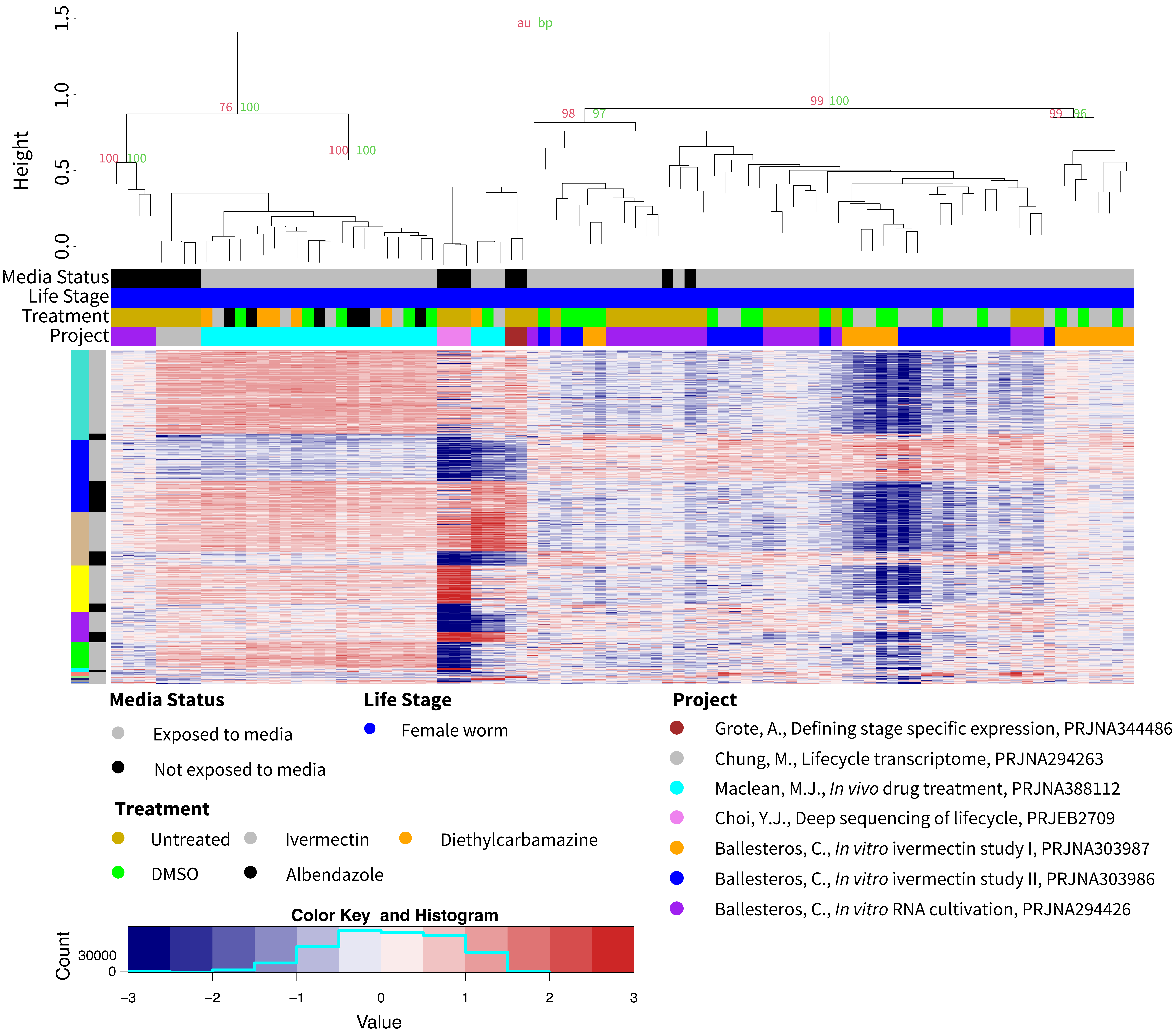

Height
0.0
0.5
1.0
1.5
au bp
76 100
100 100
100 100
98 97
99 100
99 96
Media Status
Life Stage
Treatment
Project
Media Status
Exposed to media
Not exposed to media
Life Stage
Female worm
Treatment
Untreated
Ivermectin
Diethylcarbamazine
DMSO
Albendazole
Project
Grote, A., Defining stage specific expression, PRJNA344486
Chung, M., Lifecycle transcriptome, PRJNA294263
Maclean, M.J., In vivo drug treatment, PRJNA388112
Choi, Y.J., Deep sequencing of lifecycle, PRJEB2709
Ballesteros, C., In vitro ivermectin study I, PRJNA303987
Ballesteros, C., In vitro ivermectin study II, PRJNA303986
Ballesteros, C., In vitro RNA cultivation, PRJNA294426
Color Key and Histogram
Count
30000
0
−3
−2
−1
0
1
2
3
Value
